# Hyperproduction of 3-hydroxypropionate by *Halomonas bluephagenesis*

**DOI:** 10.1038/s41467-021-21632-3

**Published:** 2021-03-08

**Authors:** Xiao-Ran Jiang, Xu Yan, Lin-Ping Yu, Xin-Yi Liu, Guo-Qiang Chen

**Affiliations:** 1 Department of Microbiology, Army Medical University, Chongqing, China; 2 School of Life Sciences, Tsinghua University, Beijing, China; 3 Center for Synthetic and Systems Biology, School of Life Sciences, Tsinghua University, Beijing, China; 4 MOE Key Lab for Industrial Biocatalysis, Department of Chemical Engineering, Tsinghua University, Beijing, China

**Keywords:** Metabolic engineering, Applied microbiology, Biopolymers

## Abstract

3-Hydroxypropionic acid (3HP), an important three carbon (C3) chemical, is designated as one of the top platform chemicals with an urgent need for improved industrial production. *Halomonas bluephagenesis* shows the potential as a chassis for competitive bioproduction of various chemicals due to its ability to grow under an open, unsterile and continuous process. Here, we report the strategy for producing 3HP and its copolymer poly(3-hydroxybutyrate-co-3-hydroxypropionate) (P3HB3HP) by the development of *H. bluephagenesis*. The transcriptome analysis reveals its 3HP degradation and synthesis pathways involving endogenous synthetic enzymes from 1,3-propanediol. Combing the optimized expression of aldehyde dehydrogenase (AldD_Hb_), an engineered *H. bluephagenesis* strain of whose 3HP degradation pathway is deleted and that overexpresses alcohol dehydrogenases (AdhP) on its genome under a balanced redox state, is constructed with an enhanced 1.3-propanediol-dependent 3HP biosynthetic pathway to produce 154 g L^−1^ of 3HP with a yield and productivity of 0.93 g g^−1^ 1,3-propanediol and 2.4 g L^−1^ h^−1^, respectively. Moreover, the strain could also accumulate 60% poly(3-hydroxybutyrate-co-32–45% 3-hydroxypropionate) in the dry cell mass, demonstrating to be a suitable chassis for hyperproduction of 3HP and P3HB3HP.

## Abstract (web summary)

3-Hydroxypropionic acid (3HP) is an important platform chemical. Here, the authors engineer *Halomonas bluephagenesis* by deleting newly identified degradation pathway and balancing redox state to achieve high level production of 3HP and its copolymer under open and unsterile conditions.

## Introduction

3-Hydroxypropionic acid (3HP) is a platform chemical for the synthesis of C3-based chemicals, including acrylic acid, malonic acid, acrylamide, acrylonitrile (ACN), and poly(3-hydroxypropionate) (P3HP) based polymers^1–4^. The chemical synthesis of 3HP is not suitable for industrial production owing to the high cost of the precursors and processes, and environmental incompatibility^5^. Since biosynthesis is sustainable and environmental friendly, it is considered a better choice for improved 3HP industrial production. Synthetic pathways were designed for producing 3HP from bio-renewable sources such as glucose, glycerol, or CO_2_^6–9^. However, its biosynthesis has been reported to have poor productivity^6^. Glucose utilizing pathways based on malonyl-CoA or β-alanine has been constructed for 3HP production^10–12^, but the complexity of manipulating and regulating multiple genes has proven to be a challenge for enhanced 3HP production^13^. Instead, glycerol, a byproduct of biodiesel, is more favorable. The glycerol to 3HP pathway involves glycerol dehydratase (GDHt) and aldehyde dehydrogenase (ALDH), converting glycerol to 3-hydroxypropionaldehyde and then 3HP, respectively^14,15^.

Traditional microbial chassis such as *Escherichia coli* or *Corynebacterium glutamicum* were reported to have the final 3HP concentration of approximately 70 g L^−1^ ^16,17^. *Klebsiella pneumoniae*, which naturally produces vitamin B_12_ as a cofactor of GDHt, showed the highest production of 82 g L^−1^ 3HP^18–20^. However, a larger-scale production using *K. pneumoniae* was impossible because it is been known as an opportunity pathogen^13^. Therefore, to enhance the production of 3HP, we need to develop a more suitable producer as the chassis. Another issue occurs during the production of 3HP: under an aerobic condition, GDHt is deactivated combined with a downregulated expression of vitamin B_12_^21^, while under anaerobic condition, cells were grown poorly, and NAD^+^, a necessary cofactor of ALDH, has difficulty to regenerate^21–24^. The trade-off between vitamin B_12_ synthesis and NAD^+^ regeneration controls 3HP production and the key is the balance of dissolved oxygen in the cultures^23^. Pure 1,3-propanediol (PDO), obtainable from microbial process (1$ kg^−1^ on www.alibaba.com, unpurified 1,3-propanediol should have a much lower price), is a monomer of a high-performed textile material, poly(1,3-trimethylene terephthalate) (PTT)^25^. Using 1.3-propanediol as the substrate may be a possible solution^26–28^, as 1,3-propanediol can also be converted into 3HP using 1,3-propanediol oxidoreductase (PDOR) and ALDH^27^. The two NAD^+^-dependent enzymes PDOR and ALDH function well under aerobic conditions without vitamin B_12_^21^. As an example, *Gluconobacter oxydans* was reported to produce 45 g L^−1^ 3HP using these two enzymes^27^. These results paved the way by using 1,3-propanediol as the carbon source to produce 3HP.

Halophilic *Halomonas* spp. have attracted attentions from industrial biotechnology researchers^29^. They are capable of growing at high concentrations of NaCl and alkaline pH, thus, avoiding possible contamination by other microorganisms even under open unsterile conditions^30^. As a result, *Halomonas* spp.-based fermentations have reduced cost and complexity^31,32^. Pilot-scale fermentations using seawater-based artificial media under open and continuous conditions were conducted without contamination over a long period of two months^32^. Moreover, several synthetic biology and chromosome engineering methods such as CRISPR/Cas9 have been successfully applied in *Halomonas* spp., turning them into a more suitable chassis for future metabolic engineering and genome editing^33,34^. Next generation of industrial biotechnology (NGIB) has been developed based on extremophile *Halomonas* spp., allowing the production to be conducted under open, unsterile, and continuous process conditions with reduced fresh water and energy consumption, aiming for increasing economic competitiveness of current industrial biotechnology^29,35,36^.

*Halomonas bluephagenesis* TD (once termed *Halomonas* sp. TD01) is a halophile isolated from Aydingol Lake in Xinjiang Province/China, has been used as a chassis for the production of several bioplastic polyhydroxyalkanoates (PHAs), including poly-3-hydroxybutyrate (PHB), poly(3-hydroxybutyrate-*co*-4-hydroxybutyrate) (P3HB4HB) and poly(3-hydroxybutyrate-*co*-3-hydroxyvalerate) (PHBV) using various engineering approaches^37–40^. *H. bluephagenesis* is capable of growing at NaCl concentrations of 20–150 g L^−1^ with an optimal concentration of 40–60 g L^−1^. The strain can be grown at a pH ranging 5.0–11.0, the optimum of which is 8.5–9.0. However, as a promising biosynthetic chassis, *H. bluephagenesis* should further expand its product diversity to create more industrial value^41^. The unique growth condition of high osmotic pressure for *H. bluephagenesis* is beneficial for producing organic acid at a high concentration^35^. The organic acids produced are in the form of sodium salt under alkaline culture conditions, preventing the formation of toxic undissociated organic acids during production^42,43^. It is therefore reasonable to expect that *H. bluephagenesis* can be a suitable organic acid producer such as 3-hydroxypropionic acid (3HP).

Here, we show that a coherent bioprocess based on halophilic *H. bluephagenesis* for production of 3HP and its copolymer poly(3-hydroxybutyrate-*co*-3-hydroxypropionate) (P3HB3HP) by identifying and deleting 3HP degradation pathway, screening and optimizing endogenous biosynthetic enzymes, engineering genomic expression of 3HP synthesis pathway and balancing redox states. The engineered *H. bluephagenesis* strain produces a higher titer of 3HP with 1,3-propanediol as a carbon source under open and unsterile fed-batch fermentation.

## Results

### Identification of 3HP degradation pathway in *H. bluephagenesis*

**Fig. 1:**
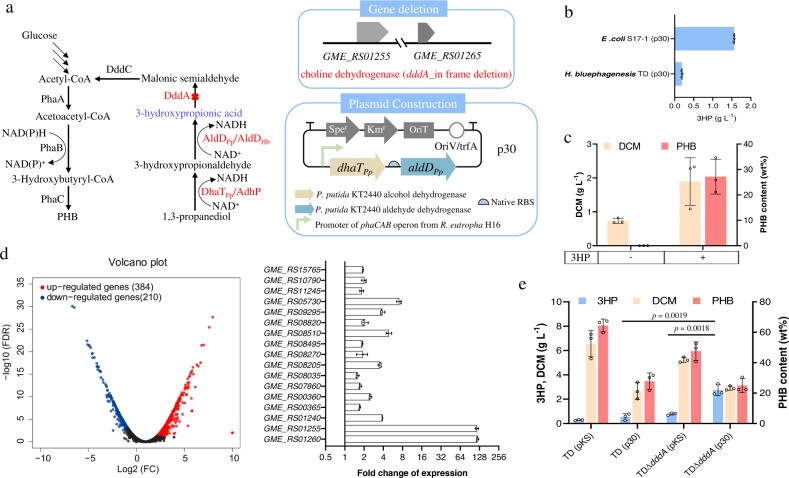
Metabolic engineering of *H. bluephagenesis* for 3HP production. **a** Overall strategies for 3HP production involving a combination of marker-free *dddA* deletion and plasmid-expressing *dhaT*_*Pp*_, *aldD*_*Pp*_. Red color “X” indicates inactivation of metabolic pathways. Gene *dhaT*_*Pp*_ encodes *P. putida* KT2440 alcohol dehydrogenase, *aldD*_*Pp*_ encodes *P. putida* KT2440 aldehyde dehydrogenase, *adhP* encodes *H. bluephagenesis* alcohol dehydrogenase, *aldD*_*Hb*_ encodes *H. bluephagenesis* aldehyde dehydrogenase. DddA putative 3-hydroxypropionate dehydrogenase in *H. bluephagenesis*, DddC CoA-acylating methylmalonate-semialdehyde dehydrogenase in *H. bluephagenesis*, PhaA 3-ketothiolase, PhaB acetoacetyl-CoA reductase, PhaC PHA synthase. **b** 3HP production by *E. coli* and *H. bluephagenesis* harboring plasmid p30, respectively. **c** PHB production by *H. bluephagenesis* cultured on 5 g L^−1^ 3HP as a sole carbon source. **d** Influence of 3HP on cell metabolism at the transcriptional level in *H. bluephagenesis*. Volcano plot and fold change of gene expression of differentially expressed gene (DEG) distribution. A corrected *P*-value of 0.05 and log2 (fold change) of 1 were set as the thresholds for significantly differential expression. Red, upregulated genes; blue, downregulated genes. qRT-PCR analysis of several putative upregulated dehydrogenases expression levels in the 3HP containing cultures compared to that of the glucose-containing ones. **e** 3HP and PHB production by *H. bluephagenesis* TDΔ*dddA* harboring plasmid p30. Cells were grown in a defined minimal medium supplemented with 30 g L^−1^ glucose and 10 g L^−1^ 1,3-propanediol. All titers were obtained after 48 h cultivation at 200 r.p.m. at 37 °C. The initial pH of all shake-flask studies was 9. All data represent the mean of *n* = 3 biologically independent samples and error bars show s.d. Two-tailed Student’s *t*-tests were performed to determine the statistical significance for two-group comparisons. DCM dry cell mass, PHB polyhydroxybutyrate.

Due to the high osmotic pressure tolerance, *H. bluephagenesis* is an ideal chassis for the extracellular production of small molecules with a high concentration in the cultures. The extracellular 3HP tolerance has been demonstrated by *H. bluephagenesis*, further proving its potential as a microbial industrial chassis for 3HP (Supplementary Fig. 1). To verify the feasibility of 3HP production by *H. bluephagenesis*, genes *dhaT*_*Pp*_ and *aldD*_*Pp*_ from *Pseudomonas putida* KT2440 encoding 1,3-propanediol dehydrogenase and aldehyde dehydrogenase, were constructed in plasmid p30 and overexpressed in *E. coli* S17-1 and *H. bluephagenesis*, respectively (Fig. 1a). The recombinant *E. coli* S17-1 (p30) produced 1.5 g L^−1^ and *H. bluephagenesis* (p30) produced 0.2 g L^−1^ 3HP when grown in conical flasks. The former strain was cultured in LB medium supplemented with 30 g L^−1^ glucose and 10 g L^−1^ 1,3-propanediol, and the latter was grown in the defined minimal medium supplemented with 30 g L^−1^ glucose and 10 g L^−1^ 1,3-propanediol (Fig. 1b). Recombinant *H. bluephagenesis* (p30) accumulated 0.5 g L^−1^ PHB when 5 g L^−1^ 3HP was added as the sole carbon source (Fig. 1c). The 3HP may be degraded to acetyl-coA for cell growth and PHB accumulation, indicating that *H. bluephagenesis* is able to utilize 3HP as a carbon source (Fig. 1a). This result suggests that *H. bluephagenesis* contains a 3HP degradation pathway.

To investigate the key genes in the 3HP degradation pathway, transcriptome analysis of *H. bluephagenesis* was performed using the cells grown under culture conditions with and without 3HP as the carbon source, respectively. A volcano plot illustrates the distribution of differentially expressed genes (DEGs) (Fig. 1d). Overall, when 3HP was used as the sole carbon source for growth, 384 genes in the cells were upregulated while 210 were downregulated compared with glucose utilized as a sole carbon source (Fig. 1d and Supplementary Data 2). Possibly similar to the catabolic operons, the addition of 3HP could activate its degradation pathway. Several putative upregulated dehydrogenases were thus selected for qRT-PCR verification. Among them, *GME_RS01260* encoding choline dehydrogenase (RefSeq. Accession: WP_009721523) and *GME_RS01255* encoding CoA-acylating methylmalonate-semialdehyde dehydrogenase (RefSeq. Accession WP_009721522), were upregulated by 119-folds and 116-folds, respectively (Fig. 1d). Choline dehydrogenase is named DddA due to its high homology with the DddA of *Halomonas* sp. HTNK1^44^ (Fig. 1a). Deletions of putative 3-hydroxypropionate dehydrogenase (HpdH) and putative 3-hydroxyisobutyrate dehydrogenase (HbdH-4) in *Pseudomonas denitrificans* eliminate its ability to degrade 3HP^45–47^. Cell growth was found to be unaffected while 3HP could no longer be utilized as the sole carbon source after the *dddA* deletion (Supplementary Figs. 2 and 3). The *dddA* gene deletion in recombinant *H. bluephagenesis* TDΔ*dddA* (p30) could increase the production with 2.7 g L^−1^ 3HP and 0.7 g L^−1^ PHB when grown in conical flasks (Fig. 1e).

The toxicity of 3-hydroxypropionaldehyde (3HPA), an intermediate for 3HP biosynthesis, negatively affects cell growth and 3HP production. Expression modules were designed to prevent the possible intracellular accumulation of 3HPA using optimized promoter, ribosome binding site (RBS) sequence of *aldD*_*Pp*_, and shifting the order of *aldD*_*Pp*_ and *dhaT*_*Pp*_ operon (Supplementary Fig. 4a). Plasmid p31 was constructed replacing the original RBS with a stronger one; p32 contains changing order of *aldD*_*Pp*_ and *dhaT*_*Pp*_ operon; p33 and p34 based on p32 were constructed with a stronger RBS and promoter, respectively.

qRT-PCR was performed to further confirm the enhanced expression level of *aldD*_*Pp*_ gene under the P_Porin_ promoter^48^. Recombinant strains were cultured in conical flasks and grown for 6, 12, 24, 36, and 48 h, respectively. The results showed that the expression level of *aldD*_*Pp*_ in *H. bluephagenesis* TDΔ*dddA* harboring p34 was higher compared to that of *H. bluephagenesis* TDΔ*dddA* (p32) during the early growth phase, whereas an opposite trend was revealed at the late growth period (Supplementary Fig. 4b). However, the enzyme activity of AldD_Pp_ was observed to decrease over time, while the activity of DhaT_Pp_ has shown an increase up to 24 hours (Supplementary Fig. 5). Additionally, we compared the enzyme activity of AldD_Pp_ in recombinant *H. bluephagenesis* TDΔ*dddA* harboring p32-p34 deleted with *dhaT*_*Pp*_ gene. The activity of AldD_Pp_ was increased using the optimized promoter or ribosome binding site (RBS) sequence of *aldD*_*Pp*,_ even though the yield of 3HP did not increase significantly (Supplementary Fig. 6a, b). In general, manipulation of *aldD*_*Pp*_ expression level showed no enhancement on 3HP production (Supplementary Fig. 6a). This may be due to the low activity of DhaT_Pp_, which leads to the weak production of toxic 3HPA intermediate. Hence, even though the poor expression of *aldD*_*Pp*_, it was enough for consuming the produced 3HPA and avoiding its accumulation. It was also shown that the specific activity of DhaT_Pp_ was lower than that of 1,3-propanediol oxidoreductase (DhaT_Kp_) in *Klebsiella pneumoniae* DSM 2026 as reported^49^ (Supplementary Fig. 6c), indicating an insufficient supply of 3HPA, which limits 3HP biosynthesis. To develop microbial cell factories for efficient 3HP production, efficient enzyme systems must be constructed^50^.

### Analysis of endogenous enzymes converting 1,3-propanediol to 3HP

A trace amount of 3HP was detected in the *H. bluephagenesis* TDΔ*dddA* harboring the empty plasmid as a control, indicating the existence of endogenous activity of native 3HP synthetic enzymes in *H. bluephagenesis* (Fig. 1e). To analyze the endogenous synthesis pathway of 3HP, a protein homologous to the aldehyde dehydrogenase AldD_Pp_ was found via in silico analysis of the genome sequence of *H. bluephagenesis*, namely, *GME_RS00360*, which was annotated as the putative *aldD*_*Hb*_ gene encoding a 506-amino-acid polypeptide. An overall 80% amino acid identity was shown between AldD_Hb_ (RefSeq. Accession: WP_009721344) and the AldD_Pp_ from *P. putida* KT2440 after BLAST analysis.

**Fig. 2:**
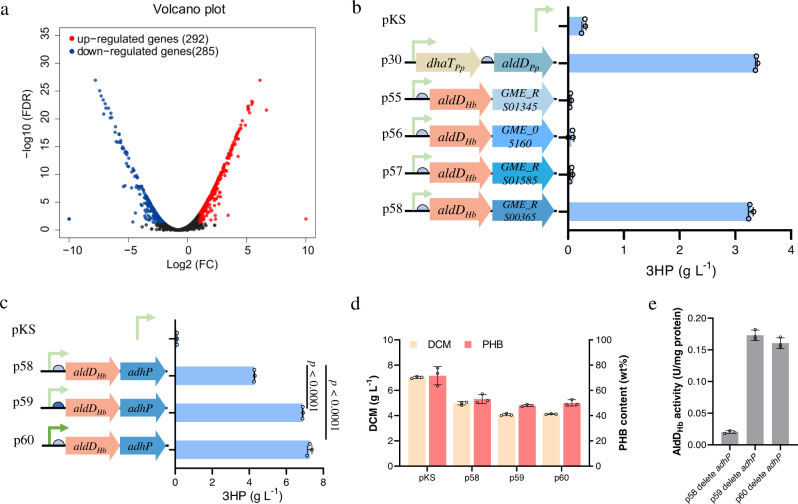
Selection of endogenous enzymes catalyzing 1,3-propanediol to 3HP biosynthesis. **a** Influence of 1,3-propanediol on cell metabolism at the transcriptional level in *H. bluephagenesis* TDΔ*dddA*. Volcano plot and fold change of gene expression of differentially expressed gene (DEG) distribution. A corrected *P*-value of 0.05 and log2 (fold change) of 1 were set as the thresholds for significantly differential expression. Red, upregulated genes; blue, downregulated genes. **b** 3HP production by *H. bluephagenesis* TDΔ*dddA* overexpressing different biosynthetic genes using 1,3-propanediol as a carbon source. Plasmid pKS and p30 were used as the negative and positive controls, respectively. Gene *dhaT*_*Pp*_ encodes *P. putida* KT2440 alcohol dehydrogenase, *aldD*_*Pp*_ encodes *P. putida* KT2440 aldehyde dehydrogenase, *aldD*_*Hb*_ encodes *H. bluephagenesis* aldehyde dehydrogenase, *GME_RS01345* encodes NAD(P)-dependent alcohol dehydrogenase; *GME_05160* encodes zinc-binding alcohol dehydrogenase, *GME_RS01585* encodes zinc-binding dehydrogenase, *GME_RS00365* encodes alcohol dehydrogenase AdhP. **c** 3HP production and **d** cell growth (DCM) and PHB content under optimal expression levels of aldehyde dehydrogenase. A stronger RBS was cloned instead of the original construct p58 to form plasmid p59. A stronger promoter Pporin replaced the original construct p58 to generate plasmid p60 (Supplementary Table 5). Cells were grown in the defined minimal medium supplemented with 30 g L^−1^ glucose, 10 g L^−1^ 1,3-propanediol, and 3 g L^−1^ acetic acid. All titers were obtained after 48 h cultivation at 200 r.p.m. and 37 °C. Initial pH of all shake-flask studies was 9. Two-tailed Student’s *t*-tests were performed to determine the statistical significance for two-group comparisons. **e** The enzyme activity of AldD_Hb_ was assayed using the recombinant *H. bluephagenesis* TDΔ*dddA* harboring p58-p60 deleted with *adhP* gene cultivated in 60-LB medium for 24 h. The comparison of activities was conducted using a crude extract. All data represent the mean of *n* = 3 biologically independent samples and error bars show s.d.

Subsequently, an transcriptome analysis of *H. bluephagenesis* was performed using cells grown with or without 1,3-propanediol in culture media containing 30 g L^−1^ glucose to investigate alcohol dehydrogenases in *H. bluephagenesis*. The volcano plot illustrates the distribution of differentially expressed genes (DEGs) (Fig. 2a). Overall, 292 genes were upregulated while 285 were downregulated in the presence of 1,3-propanediol in the cultures (Fig. 2a and Supplementary Data 3). Several putative upregulated alcohol dehydrogenases were examined, including *GME_RS01345, GME_05160, GME_RS01585* and *GME_RS00365* encoding NAD(P)-dependent alcohol dehydrogenase (RefSeq. Accession: WP_009721539.1), zinc-binding alcohol dehydrogenase (GenBank Accession: EGP20677.1), zinc-binding dehydrogenase (RefSeq. Accession: WP_009721587.1), and alcohol dehydrogenase AdhP (RefSeq. Accession: WP_039868491.1), respectively (Fig. 2b).

In order to overexpress the mentioned genes encoding putative alcohol dehydrogenases, four related plasmids were constructed combined with the gene *aldD*_*Hb*_ encoding aldehyde dehydrogenase, and *H. bluephagenesis* TDΔ*dddA* was transformed using the resulting plasmids, respectively (Fig. 2b). The result showed only one strain harboring *GME_RS00365* gene *adhP* was able to produce 3.3 g L^−1^ 3HP (Fig. 2b). Thus, the gene *adhP* was identified to encode the 1,3-propanediol dehydrogenase with relatively high activity to convert 1,3-propanediol to 3HP.

An expression module, which does not hamper the cell growth during the 3HP production, was designed by optimizing the expression levels of *aldD*_*Hb*_ encoding aldehyde dehydrogenase (Fig. 2c). The *H. bluephagenesis* TDΔ*dddA* harboring p59 equipped with a stronger RBS and p60 with a stronger promoter, respectively, achieved a higher yield of ~7 g L^−1^ 3HP, compared to the control *H. bluephagenesis* TDΔ*dddA* harboring p58 without a stronger promoter or RBS (Fig. 2c). Moreover, polyhydroxybutyrate (PHB) contents produced by *H. bluephagenesis* TDΔ*dddA* (p58), *H. bluephagenesis* TDΔ*dddA* (p59) and *H. bluephagenesis* TDΔ*dddA* (p60) were similar (Fig. 2d). The above results demonstrated that the *aldD*_*Hb*_ upregulated expression level could enhance 3HP production. In addition, we compared the enzyme activity of AldD_Hb_ in the recombinant *H. bluephagenesis* TDΔ*dddA* harboring p58, p59 or p60 deleted with the *adhP* gene (Fig. 2e). Consistent with the significant increase of 3HP yield, the enzyme activity of AldD_Hb_ was significantly increased by optimizing the promoter or ribosome binding site (RBS) sequence of *aldD*_*Pp*_ (Fig. 2e). Since 1,3-propanediol was almost completely consumed while glucose was still available at 48 h of cultivation when 30 g L^−1^ glucose and 10 g L^−1^ 1,3-propanediol were added to cultures, a higher final 3HP titer is expected when there are more 1,3-propanediol and less glucose (Supplementary Fig. 7).

### Selection of efficient enzymes for biosynthesis of 3HP in *H. bluephagenesis*

**Fig. 3:**
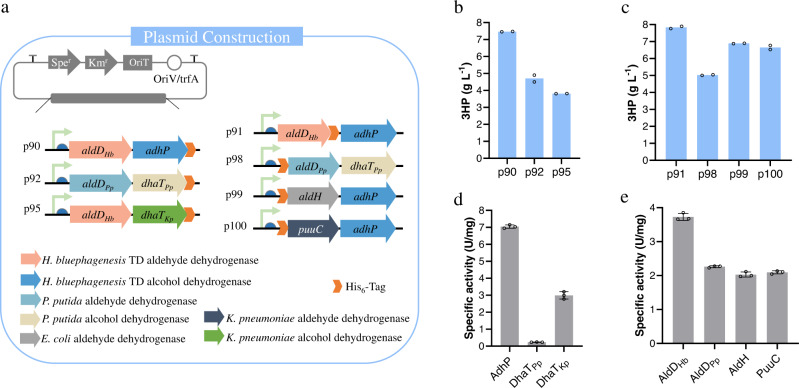
Selection of efficient enzymes for biosynthesis of 3HP in *H. bluephagenesis*. **a** Construction of the plasmids to express enzymes from different species. Following plasmids were constructed: plasmid p90 expressing *adhP* gene with a His-tag on C-terminal, p91 expressing *aldD*_*Hb*_ gene with a His-tag on C-terminal, p92 expressing *dhaT*_*Pp*_ gene with a His-tag on C-terminal and p98 expressing *aldD*_*Pp*_ gene with a His-tag on N-terminal. The *aldH* gene, with a His-tag on N-terminal, was amplified via PCR using *E. coli* genomic DNA as a template and cloned into the p58 by replacing the *aldD*_*Hb*_ gene. This plasmid is labeled as p99. The *dhaT*_*Kp*_ or *puuC* gene, with a His-tag, was amplified via PCR using *K. pneumoniae* DSMZ 2026 genomic DNA as a template and cloned into the p58 by replacing the *adhP* or *aldD*_*Hb*_ gene. These plasmids were labeled as p95 and p100. 3HP production of different alcohol dehydrogenases (**b**), and different aldehyde dehydrogenases (**c**). Cells were grown in the defined minimal medium containing 20 g L^−1^ glucose, 20 g L^−1^ 1,3-propanediol, and 3 g L^−1^ acetic acid. All titers were obtained after 48 h cultivation at 200 r.p.m. and 37 °C. The initial pH of all shake-flask studies was 9. All data represent the mean of *n* = 2 biologically independent. The comparation of the various alcohol dehydrogenases oxidative activities (**d**), and various aldehyde dehydrogenases activities (**e**) in a purified enzyme. All data represent the mean of *n* = 3 biologically independent samples and error bars show s.d.

With the successful establishment of 3HP production platform based on *H. bluephagenesis*, 3HP production needed further improvement. The 3HP biosynthetic pathway from 1,3-propanediol comprises two parts, including the formation of 3HPA and subsequent conversion of 3HPA to 3HP (Fig. 1a). To select the efficient enzyme combinations for the 3HP production via 1,3-propanediol oxidoreductase and aldehyde dehydrogenase, different plasmids were constructed to express variants of different pathway components. Specifically, aldehyde dehydrogenase AldH (RefSeq. Accession: WP_001009090.1) of *E. coli* MG1655, 1,3-propanediol oxidoreductase DhaT_Kp_ (GenBank AAP97875.1) and aldehyde dehydrogenase PuuC (RefSeq. Accession: WP_004224052.1) of *K. pneumoniae* were selected (Fig. 3a)^49,51,52^. In the enzyme-screening process, we first compared the efficiency of the selected 1,3-propanediol oxidoreductase encoding genes in combination with *aldD*_*Hb*_ (plasmids p90 and p95). With the co-overexpression of *adhP*, the resultant strain *H. bluephagenesis* TDΔ*dddA* (p90) was shown to have the highest 3HP production (Fig. 3b). Subsequently, the selected aldehyde dehydrogenases encoding genes were evaluated based on *adhP* (plasmids p91, p99, and p100). The *aldD*_*Hb*_ still outperformed its counterparts, producing 3HP at a titer of 7.8 g L^−1^ (*H. bluephagenesis* TDΔ*dddA* (p91), Fig. 3c). However, *H. bluephagenesis* TDΔ*dddA* (p105) containing the *K. pneumoniae* PuuC combination with *K. pneumoniae* DhaT_Kp_ only generated 3HP at a titer of 2 g L^−1^ (Supplementary Fig. 8a).

Enzyme activities in the recombinant *H. bluephagenesis* TDΔ*dddA* overexpressing the three different alcohol dehydrogenases (AdhP, DhaT_Pp_, and DhaT_Kp_) and four different aldehyde dehydrogenases (AldD_Hb_, AldD_Pp_, AldH, and PuuC) were analyzed using their corresponding purified enzymes, respectively (Fig. 3a). The His-tagged enzymes were purified to electrophoretic homogeneity (Supplementary Fig. 9). For the oxidative activity, the specific activity of AdhP was found to be ~7.0 U mg^−1^, higher than the alcohol dehydrogenase of *P. putida* DhaT_Pp_ and *K. pneumoniae* DhaT_Kp_ (Fig. 3d). The aldehyde dehydrogenase enzyme is in general presented as a tetramer. Its C terminus has been known to play an important role in the assembly, and thus a His-tag was introduced to the N terminus. However, the production of 3HP was significantly reduced when the His-tag was introduced to the N terminus of AldD_Hb_ (Supplementary Fig. 8b). Thus, we introduced the His-tag to the C terminus of AldD_Hb_. The specific activity of AldD_Hb_ was ~3.7 U mg^−1^, the highest among all the aldehyde dehydrogenases (Fig. 3e). The AdhP and AldD_Hb_ enzymes kinetic properties of *K*_cat_ and *K*_m_ were characterized (Table 1). The half-saturation constant (*K*_m_) of AldD_Hb_ was low at 0.05 mM, indicating that the AldD_Hb_ has a very high affinity for the substrate 3HPA, and the catalytic efficiency (*k*_cat_/*K*_m_) of the AldD_Hb_ was 100 times higher than that of PuuC at the same condition (Supplementary Table 3). Thus, AldD_Hb_ and AdhP were considered as the efficient enzymes for biosynthesis of 3HP by *H. bluephagenesis*.

**Table 1:** Kinetic parameters of AdhP and AldD_Hb_.

Enzymes	*K*_m_ (mM)	*K*_cat_ (s^−1^)	*K*_cat_/*K*_m_ (s^−1^ mM^−1^)
AdhP^a^	35.41 ± 1.525	4.11 ± 0.05	0.12 ± 0.004
AdhP^b^	1.75 ± 0.278	19.39 ± 0.62	11.19 ± 1.304
AldD_Hb_^c^	0.05 ± 0.002	3.38 ± 0.09	67.45 ± 0.797

Each value represents the mean ± standard deviation in triplicate experiments.

^a^Assay conditions: 100 mM potassium carbonate buffer (pH 9.0) containing 30 mM ammonium sulfate; 2 mM, NAD^+^; 9.7 μg mL^−1^, AdhP; 1,3-propanediol concentration was varied from 1.17 to 600 mM; temperature was set at 37 °C.

^b^Assay conditions: 50 mM Tris-HCl buffer (pH 7.0) containing 0.1 mM NADH; 0.36 μg mL^−1^, AdhP; 3HPA concentration was varied from 0.01 to 20 mM; temperature was set at 37 °C.

^c^Assay conditions: 50 mM, potassium phosphate buffer (pH 8.0) containing 1.0 mM DTT; 2 mM, NAD^+^; 5.7 μg mL^−1^, AldD_Hb_; 3HPA concentration was varied from 0.02 to 15 mM; temperature was set at 37 °C.

### Combinatorial optimization for increasing the production of 3HP

**Fig. 4:**
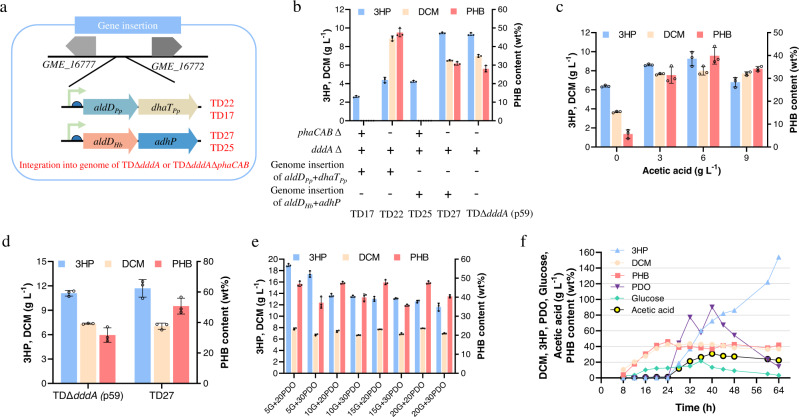
Combinatorial optimization to increase 3HP production. **a** Genome engineering strategy and genetic constructs used for genomic integration. **b** Genome-overexpression and deletion of enzymes in the 1,3-propanediol to 3HP biosynthetic pathway enhanced 3HP production. Gene *dddA* and PHB synthesis operon *phaCAB* encoding 3HP degradation enzyme and PHB synthesis enzymes were deleted, respectively. 1,3-propanediol to 3HP biosynthetic pathway of *P. putida* KT2440 and *H. bluephagenesis* were inserted into *H. bluephagenesis* TDΔ*dddA* and *H. bluephagenesis* Δ*dddA*Δ*phaCAB*, respectively. Cells were grown in the defined minimal medium supplemented with 20 g L^−1^ glucose, 20 g L^−1^ 1,3-propanediol, and 3 g L^−1^ acetic acid. **c** Characterization of 3HP and PHB produced by metabolically engineered *H. bluephagenesis* TD27 cultured at gradient concentrations of acetic acid to balance the redox state. Cells were grown in the defined minimal medium containing 20 g L^−1^ glucose, 20 g L^−1^ 1,3-propanediol, and gradient concentrations of acetic acid (g L^−1^) 0, 3, 6, and 9, respectively. The pH was adjusted to 9 after incubation for 24 h. **d** Characterization of 3HP and PHB produced by metabolically engineered *H. bluephagenesis* TD27 cultured at a modified minimal medium containing different phosphate buffers to balancing the pH fluctuations. Cells were grown with 20 g L^−1^ glucose, 20 g L^−1^ 1,3-propanediol, and 6 g L^−1^ acetic acid. All titers were obtained after 48 h cultivation at 200 r.p.m. and 37 °C. **e** 5, 10, 15, and 20 g L^−1^ glucose (5G, 10G, 15G, and 20G) were co-fed with 20 g L^−1^ 1,3-propanediol (20PDO) or 30 g L^−1^ 1,3-propanediol (30PDO), respectively, to cultures of *H. bluephagenesis* TD27 grown in a modified minimal medium containing 6 g L^−1^ acetic acid. **f** Time profiles of cell growth (DCM), PHB content, and concentrations of carbon sources (glucose, 1,3-propanediol, acetic acid), and 3HP production during the fed-batch culture of *H. bluephagenesis* TD27. 60 g L^−1^ 1,3-propanediol and 6 g L^−1^ acetic acid were added at 24, 32 and 38 h during fed-batch culture, respectively. For the shake-flask cultivation, The initial pH of all shake-flask studies was 9. All data represent the mean of *n* = 3 biologically independent samples and error bars show s.d.

To stabilize microbial production, it is critical to obtain stable gene expressions in *H. bluephagenesis*. This is commonly accomplished via genomic integration of genes of interest. To efficiently manipulate relevant genes, a marker-free CRISPR/Cas9 genome-editing technology was employed^33^, allowing the construction of a 3HP biosynthetic pathway for genome integration (Fig. 4a). The best-performing gene combination, *aldD*_*Hb*_ with *adhP* containing promoter of *phaCAB* operon from *Ralstonia eutropha* H16 and a stronger RBS, were integrated into the chromosome of *H. bluephagenesis* for evaluation on 3HP production (Fig. 4a). The genomic integration of the 3HP biosynthetic pathway does not negatively impact cell growth (Supplementary Fig. 10). As a result, a significant increase of 3HP production was observed in recombinant *H. bluephagenesis* TD22 and *H. bluephagenesis* TD27 containing intact PHB production pathway when compared with its PHB synthesis operon *phaCAB* deleted strains *H. bluephagenesis* TD17 and *H. bluephagenesis* TD25, respectively, indicating that the PHB accumulation could promote 3HP synthesis. Moreover, *H. bluephagenesis* TD27 harboring genome-expressing 3HP endogenous synthetic pathway produced ~9 g L^−1^ 3HP, slightly higher than that synthesized by the plasmid-expressing *H. bluephagenesis* TDΔ*dddA* (p59). Therefore, *H. bluephagenesis* TD27 was taken as the platform for further enhanced 3HP production from 1,3-propanediol (Fig. 4b).

1 M 3HP can be generated from 1 M 1,3-propanediol, accompanied by the generation of 2 M NADH (Fig. 1a). However, an abnormal increase of NADH could lead to cell injury, reduced glycolysis, and TCA cycle^53^. It has been reported that surplus NADH can be used for the reductive metabolism of acetic acid to ethanol in some *Clostridium* species^54^. Acetate is a cost-effective carbon source, which can be obtained from carbon dioxide and hydrolysis or pyrolysis of lignocellulosic biomass like corn stover. Gradient concentrations of acetic acid (0, 3, 6, and 9 g L^−1^) were co-fed to the culture medium of *H. bluephagenesis* TD27 with 20 g L^−1^ glucose and 20 g L^−1^ 1,3-propanediol, respectively (Fig. 4c). The addition of 3 g L^−1^ acetic acid enhanced 3HP production to 8.6 g L^−1^ in shake-flask studies, representing a 35% increase compared to cultures in the absence of acetic acid. As a result, cell growth in terms of dry cell mass (DCM) and PHB content (PHB/DCM) increased 108 and 455%, respectively. The addition of 6 g L^−1^ acetic acid resulted in the highest 3HP, dry cell weight (DCM), and PHB content, reaching approximately 9.3 g L^−1^ 3HP, 8 g L^−1^ DCM, and 40% PHB/DCM (Fig. 4c). The addition of 6 g L^−1^ acetic acid also resulted in a 1.5-fold increase in a total amount of NAD^+^ and NADH and twofold increase in NAD^+^/NADH ratio compared with the result of 0 g L^−1^ acetic acid (Supplementary Fig. 11). Since PhaB of *H. bluephagenesis* is dependent on NADH for PHB synthesis, the synthesis of PHB consumes NADH^55^. The supply of acetic acid can increase acetyl-CoA pool. The excess NADH resulted from 3HP formation via 1,3-propanediol oxidation can be balanced by accelerating PHB synthesis via a larger acetyl-CoA pool, this improves not only the PHB synthesis but also glycolysis, TCA cycle, and 3HP synthesis (Supplementary Fig. 12). Therefore, l-aspartate as the precursor for the de novo biosynthesis of NAD^+^ was possibly improved. As a result, both the total amount of NAD^+^, NADH, and the NAD^+^/NADH ratio were increased.

The pH in the cultures decreased from 9 to 5–6 after 24 h cultivation as the increase of 3HP in shake-flask inhibits cell growth and 3HP production. To stabilize pH above 7 after 24 h cultivation via improving the buffer capacity in the medium, the phosphate buffer was changed to 1.4% K_2_HPO_4_·3H_2_O and 0.52% KH_2_PO_4_ in a modified minimal medium. This generated 12 g L^−1^ 3HP, 7 g L^−1^ DCM and 50% PHB/DCM from *H. bluephagenesis* TD27 grown in shake flasks, this was comparable to that of plasmid-expressing *H. bluephagenesis* TDΔ*dddA* (p59) (Fig. 4d), along with an over 90% conversion ratio of 1,3-propanediol to 3HP (Supplementary Fig. 13).

The glucose level should be reduced to allow the minimum amount of glucose for efficient production of 3HP. Gradient concentrations of glucose (5, 10, 15, and 20 g L^−1^) were co-fed with 20 or 30 g L^−1^ 1,3-propanediol, respectively, to cultures of *H. bluephagenesis* TD27 (Fig. 4e). The addition of 5 g L^−1^ glucose and 20 g L^−1^ 1,3-propanediol resulted in ~18.9 g L^−1^ 3HP, 7.8 g L^−1^ DCM, and 47% PHB/DCM (Fig. 4e). In the presence of 5 g L^−1^ glucose and 20 g L^−1^ 1,3-propanediol, 3.5 g L^−1^ residual 1,3-propanediol together with 0.2 g L^−1^ acetic acid were found in the culture together with less than 0.1 g L^−1^ residual glucose in the cultures of *H. bluephagenesis* TD27, indicating that the reduction of glucose enhanced 3HP production (Supplementary Fig. 14). An excess amount of NADH is formed during glycolysis and degradation of pyruvate to acetyl-CoA in the presence of sufficient glucose. The excessive NADH condition associated with 3HP formation and glycolysis can in turn inhibit glycolysis and 3HP synthesis (Supplementary Fig. 12). Thus, a reduced amount of glucose may reduce the NADH produced by glycolysis during 3HP synthesis, resulting in more conversion of 1,3-propanediol to 3HP.

*E. coli* and *H. bluephagenesis* were transformed using plasmids p30 and p59 expressing AldD_Pp_ and DhaT_Pp_ cloned from *P. putida* KT2440, AldD_Hb_ and AdhP from *H. bluephagenesis*, respectively. *E. coli* was cultured in a modified minimal medium containing 1% NaCl, and *H. bluephagenesis* in another modified minimal medium containing 6% NaCl. Recombinant *E. coli* harboring alcohol dehydrogenase (AdhP) and aldehyde dehydrogenase (AldD_Hb_) cloned from *H. bluephagenesis* produced only 0.7 g L^−1^ 3HP. In contrast, the *H. bluephagenesis* TDΔ*dddA* (p59) generated 11 g L^−1^ 3HP compared to only 5 g L^−1^ 3HP secreted by *H. bluephagenesis* TDΔ*dddA* (p30) harboring the DhaT_Pp_ and AldD_Pp_ from *P. putida* KT2440 (Supplementary Fig. 15).

To further enhance 3HP production, the fed-batch fermentation process was optimized employing various feeding strategies, including continuous or intermittent feeding strategy during the late period of growth. During the continuous feeding process, cell growth was inhibited when 1,3-propanediol and glucose were fed at 8 h. 60 g L^−1^ 3HP with <30 g L^−1^ DCM were obtained from cultures of *H. bluephagenesis* TD27 (Supplementary Fig. 16). The production of 3HP will convert a large amount of NAD^+^ into NADH during the logarithmic growth phase, which changes the redox balance. Excessive NADH generates feedback inhibition to glycolysis, negatively affecting cellular catabolism and growth. In addition, the enzyme activity of AldD_Hb_ was observed to decrease over time, and the activity of AdhP was observed to increase up to 24 h (Supplementary Fig. 17). When 60 g L^−1^ 1,3-propanediol was added at 24, 28, and 32 h during fed-batch fermentation process to separate cell growth and 3HP production, the engineered *H. bluephagenesis* TD27 produced 154 g L^−1^ 3HP with a yield and productivity of 0.93 g g^−1^ 1,3-propanediol and 2.4 g L^−1^ h^−1^, respectively (Fig. 4f).

### Engineering *H. bluephagenesis* TD27 for productions of 3HP and its copolymers

**Fig. 5:**
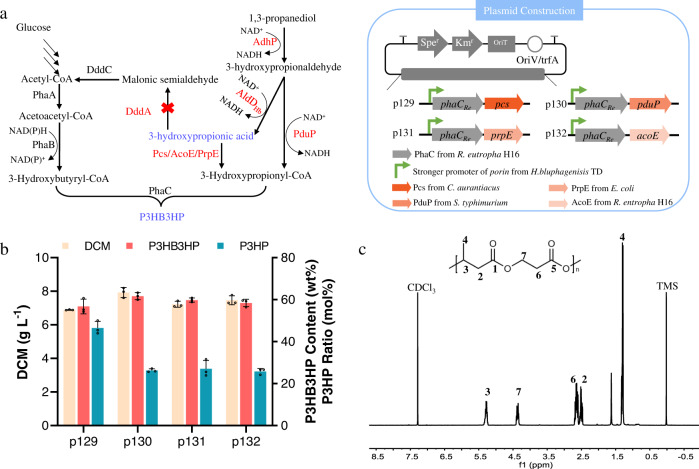
Enzyme screening for improving biosynthesis of P3HB3HP by engineered *H. bluephagenesis* TD27. **a** Strategies for microbial co-production of 3HP and P3HB3HP involving a combination of genome-based gene expression and plasmid-based gene expression. Red color “X” indicates the inactivation of metabolic pathways. Gene *adhP* encodes 1,3-propanediol dehydrogenase from *H. bluphagenesis*, *aldD*_*Hb*_ encodes aldehyde dehydrogenase from *H. bluphagenesis*, *pcs* encodes coenzyme A ligase domain from *Chloroflexus aurantiacus*, *pduP* encodes propionaldehyde dehydrogenase from *Salmonella typhimurium*, *prpE* encodes propionyl-CoA synthase from *E. coli*, *acoE* encodes acetyl-CoA synthase from *Ralstonia entropha*. Genes were expressed under the control of constitutive promoters in a multiple-copy episomal plasmid pKS. **b** P3HB3HP contents and monomer compositions generated by engineered *H. bluephagenesis* TD27 overexpressing different 3-hydroxypropionyl-CoA biosynthetic genes. Cells were grown in the modified minimal medium containing 20 g L^−1^ glucose, 10 g L^−1^ 1,3-propanediol, and 6 g L^−1^ acetic acid. All titers were obtained after 48 h cultivation at 200 r.p.m. and 37 °C. The initial pH of all shake-flask studies was 9. All data represent the mean of *n* = 3 biologically independent samples and error bars show s.d. **c** ^1^H NMR of P(3HB-*co*-40% 3HP) copolymers produced by the engineered *H. bluephagenesis* TD27 overexpressing *pcs*. TMS tetramethylsilane (internal standard).

Polyhydroxybutyrate or poly-3-hydroxybutyrate (PHB), is very brittle due to its high crystallinity that limits its application. In contrast, poly-3-hydroxypropionate (P3HP) shows good combined mechanical properties including an elongation at break of more than 600% and Young′s modulus of 300 MPa–3 GPa^56^. The low melting temperature *T*_m_ of P3HP is <100 °C, which limits its stability in a higher temperature environment. On the other hand, the *T*_m_ of PHB is high which can reach 176 °C. Thus, copolymerization of 3HB and 3HP compensate for the weak properties of each homopolymers^57^. Since recombinant *H. bluephagenesis* produced 3HP efficiently, 3HP-related copolymers are expected to be produced appropriately. It was reasonable to assume that adequate 3HP-CoA and appropriate PhaC polymerase are essential for the accumulation of copolymer P3HB3HP consisting of 3-hydroxybutyrate (3HB) and 3HP, given the PHB is natively synthesized by *H. bluephagenesis* (Fig. 5a). To produce P3HB3HP, coenzyme A ligase domain (Pcs) from *Chloroflexus aurantiacus*, propionyl-CoA synthase (PrpE) from *E. coli*, acetyl-CoA synthase (AcoE) from *Ralstonia entropha*, and propionaldehyde dehydrogenase (PduP) from *Salmonella typhimurium*, were selected to generate 3HP-CoA from 3-hydroxypropionic acid and from 3-hydroxypropionaldehyde, respectively. PhaC polymerase from *R. entropha* was chosen for its strong 3HP polymerization ability when expressed in other bacteria including *E. coli* (Fig. 5a)^58^.

The strong 3HP producer *H. bluephagenesis* TD27 was then transformed with the four constructs, and P3HB3HP was successfully produced in 7 g L^−1^ DCM containing approximately 60% copolymer (Fig. 5b). *H. bluephagenesis* TD27 overexpressing Pcs and PhaC reached 45% 3HP molar ratio in the P3HB3HP content. The extracellular 3HP yield increased gradually with the increase of 1,3-propanediol concentration in the cell cultures. The P3HB3HP content and P3HP monomer composition increased to a maximum of 54 and 45%, respectively, when 12 g L^−1^ 1,3-propanediol was added to the cultures (Supplementary Fig. 18). The glucose and 1,3-propanediol concentrations were optimized to find the minimum amount of glucose and 1,3-propanediol for efficient production of P3HB3HP. The P3HP molar ratios in P3HB3HP were found to be between 32 and 40% when different concentrations of 1,3-propanediol (2, 4, and 8 g L^−1^) were added, respectively. The DCM and P3HB3HP content increased with the increase of glucose concentration (Supplementary Fig. 19). The fed-batch production of P3HB3HP allowed a 60% polymer accumulation with the P3HP ratio reaching 40% in the copolymers (Supplementary Fig. 20).

The composition of a P3HB3HP was studied using NMR (Fig. 5c). Two characteristic peaks of P3HP at *δ* = 2.59–2.70 and *δ* = 4.31–4.39 ppm (peaks 6 and 7) were identified, together with three peaks of P3HB at *δ* = 1.24–1.33, *δ* = 2.47–2.56 and *δ* = 5.23–5.35 ppm (peaks 4, 2, and 3). The molar ratio of P3HP in this copolymer has been shown can achieve approximately 40% when calculated by integrating the areas of specific related signals.

## Discussion

*H. bluephagenesis* and its recombinants have been demonstrated to be economic chassis for reducing cost for bulk chemicals^37^. A systematic metabolic engineering approach was taken to achieve efficient production by deleting the 3HP degradation pathway combined with overexpression of DhaT_Pp_ and AldD_Pp_ proteins from *P. putida* KT2440 for 3HP production in *H. bluephagenesis* grown in the presence of 1,3-propanediol (Fig. 1a). Moreover, based on the RNA-seq data, the *adhP* and *aldD*_*Hb*_ genes were chosen and overexpressed for enhanced catalytic activities toward 3HP flux (Fig. 2). The two combinations of genes along with promoter of *phaCAB* operon from *Ralstonia eutropha* H16 and stronger RBS were inserted into the genome of the *Halomonas* chassis to approach better production performance (Fig. 4a, b). Combining with balancing the intracellular redox state via acetic acid uptake, the recombinant chassis can synthesize 154 g L^−1^ 3HP during the fed-batch growth (Fig. 4f).

Since 3HP based polyhydroxyalkanoates (PHA) polyesters provide improved mechanical strengths for PHA^59^, it has become an attractive idea to make 3HP based PHA. In this study, P3HP3HB copolymer was produced based on the established 3HP synthesis pathway combined with overexpression of *pcs* and *phaC*, two heterologous genes (Fig. 5a). The resulting P3HP3HB copolymer contained a 45% P3HP molar ratio when fed with 1,3-propanediol. The P3HP ratio also can be tuned from 32 to 40% by varying the co-feeding concentrations of 1,3-propanediol and glucose. For the first time, engineered *Halomonas* sp. has been employed to produce P3HB3HP.

The selected substrate 1,3-propanediol contributed to the high 3HP titer (Fig.1a). As mentioned above, 3HP production via metabolic engineering started from 1,3-propanediol could avoid the paradox between glycerol dehydratase and aldehyde dehydrogenase activities, which generate from the imbalance of vitamin B_12_ synthesis and NAD^+^ regeneration. Under the aerobic catalysis of 1,3-propanediol oxidoreductase and aldehyde dehydrogenase, 3HP was formed via the two-step bio-catalysis (Fig. 1a)^23^. This study proposed to use 1,3-propanediol as a substrate for 3HP production. As 1,3-propanediol can be produced efficiently from glycerol, a biodiesel production byproduct under an anaerobic bioprocess, it is cost-effective^60,61^. Under the 1,3-propanediol production conditions, 3HP was outcompeted with relatively low titer due to the low aldehyde dehydrogenase enzyme activity under anaerobic conditions^6^. In this study, 1, 3-propanediol was converted into 3HP, thus bridging low-cost glycerol to high-valued 3HP efficiently. Also, fewer secondary by-products were generated in the traditional processes under aerobic fed-batch condition, especially lactic acid, which is difficult to be separated from 3HP in the culture^6^.

In this study, glucose was added to support cell growth and PHB synthesis. The synthesis of 3HP is derived from 1,3-propanediol (Supplementary Fig. 12). Since crude 1,3-propanediol (not the purified one for making polymer PTT) produced from the microbial conversion of crude glycerol (from biodiesel production) is sold on Chinese local market with a price of $600/ton, conversion of 1,3-propanediol to 3HP is almost 100%, the material cost of 3HP should be around $600/ton excluding other minimal substrate costs. Since 3HP has not been available in large scale on the market, its price is offered based on grams. This is not much value as references for bulk chemicals. However, P3HP3HB with an improved material property over the existing PHBV sold at a price of $7000/ton, should have a much high market value even though 1,3-propanediol is used as a co-substrate.

When PHB production came from consumption of glucose, 3HP yield was increased from 4.2 to 6.4 g L^−1^, and ~0.21 g L^−1^ PHB was produced from 5.8 g L^−1^ glucose (Supplementary Table 4). Theoretically, the conversion of 1 M glucose to acetyl-CoA can produce 4 M NADH, and 1 M 3HB-CoA is generated from 2 M acetyl-CoA, diverting acetyl-CoA away from the TCA cycle where much of the NADH is generated, reducing the formation of 3 M NADH. Therefore, 0.21 g L^−1^ PHB (approximately 2.44 mM 3HB-CoA) can reduce 7.32 mM NADH formation. The 3HP yield was improved by the regeneration of NAD^+^ (via consuming NADH). The excess NADH resulted from 3HP formation via the 1,3-propanediol oxidation feedback inhibits the glycolysis and 3HP production. On the other hand, PHB production from glucose diverts acetyl-CoA away from the TCA cycle. The decrease in anabolism resulting from a weakened TCA leads to the accumulation of NADPH. The increased NADPH inhibits glucose 6-phosphate dehydrogenase activity, resulting in the accumulation of glucose 6-phosphate, thus causing a lower glucose uptake rate. Therefore, the total amount of glucose used reduced 3 g L^−1^ (16.67 mM) compared with no PHB production (Supplementary Table 4), further reducing the formation of 66.68 mM NADH. In conclusion, 74 mM (66.68 plus 7.32) NAD^+^ is saved for 3HP production. Compared with no PHB production, the yield of 3HP should be increased by 3.3 g L^−1^. In fact, 3HP yield increased by 2.2 g L^−1^. This may be due to the excess NADH via the 1,3-propanediol oxidation that also feedback inhibits the 3HP production.

When PHB production from both glucose and acetic acid, 3HP yield was increased from 6.4 to 9.3 g L^−1^, and ~3.2 g L^−1^ PHB was produced from 10.5 g L^−1^ glucose and 4 g L^−1^ acetic acid (Supplementary Table 4). Theoretically, 1 M NADH is needed to produce 1 M 3HB-coA from 2 M acetic acid regardless of the ATP consumption during the conversion of acetate to acetyl-CoA. Therefore, 4 g L^−1^ acetic acid (66.67 mM) can consume 33.33 mM NADH and produce 33.33 mM 3HB-CoA (~2.9 g L^−1^ PHB). Compared with PHB production from glucose, the yield of 3HP should be increased by 1.5 g L^−1^. In fact, 3HP yield increased by 2.9 g L^−1^. This may be attributed to the utilization of ATP that can also promote the consumption of NADH by the respiratory chain. When acetate is added, the consumption of NADH improves 3HP synthesis and glycolysis by accelerating PHB synthesis from a bigger acetyl-CoA pool, the true cell mass increased from 3.5 to 4.8 g L^−1^. As a result, 3HP be produced at 9.3 g L^−1^ with the yield of 1.8 mol mol^−1^ glucose from flask scale studies (Supplementary Table 4). When the amount of glucose used was 5 g L^−1^, 3HP was produced at 18.9 g L^−1^ with the yield of 7.6 mol mol^−1^ glucose on flask scales (Supplementary Fig. 14).

Since *H*. *bluephagenesis* is able to grow under an unsterilized conditions with a high tolerance to a high concentration of salt, in this case, it is salt of 3HP, the chassis is promising for industrial production of high titer extracellular small molecules including threonine, succinate, and itaconate^62,63^. In contrast, the most commonly used bacterium, *E. coli*, demonstrated to have a growth inhibition at 10 g L^−1^ 3HP in its culture^64^. *H. bluephagenesis* has been used to produce several PHA including PHB, PHBV, and P3HB4HB. It could be further extended for more PHA synthesis since many PDORs and ALDHs possess broad substrate specificities with the potentials for synthesizing other monomers^65,66^. Poly(3-hydroxybutyrate-*co*-5-hydroxyvderate) (P3HB5HV) and Poly(3-hydroxybutyrate-*co*-6-hydroxyhexanoate) (P3HB6HHX) could also be produced by supplementing corresponding diols to cultures of *H. bluephagenesis* based on the polymer biosynthetic methods mentioned above as some broad specificity CoA transferase and PhaC have been reported^67^. Moreover, it can be assumed that pure P3HP can be produced by deleting PHB synthesis pathway. Since PhaB of *H. bluephagenesis* is dependent on NADH as a cofactor for PHB synthesis, the PHB accumulation can further consume NADH, thus facilitating further synthesis of 3HP. The 3HP monomer synthesis decreased once the deletion of PHB synthesis pathway is completed. In order to produce pure P3HP, the introduction of other metabolic pathways to regenerate NAD^+^ can promote 3HP synthesis after the loss of PHB synthesis pathways. Further investigations could generate more unusual PHA from this chassis.

In conclusion, this study established an effective 3HP production strategy using 1,3-propanediol as a substrate by halophilic chassis *H. bluephagenesis*, demonstrating this strain to be an excellent chassis. The 3HP has also been found to be able to become a monomer of the PHA copolymer P3HP3HB. *H. bluephagenesis*, as a platform for the next generation industrial biotechnology, holds a bright prospect for low-cost productions of both extracellular and intracellular products.

## Methods

### Bacterial strains and growth conditions

Strains and plasmids used in this study are listed in Supplementary Tables 1 and 2. Primers used in this study are listed in Supplementary Data 1. *Halomonas bluephagenesis* strain TD01, the wild-type isolated from Aydingol Lake of Xinjiang province in China, has been deposited in the China General Microbiological Culture Collection Center (CGMCC) under the collection number 4353. *Escherichia coli* S17-1 was used as the host for plasmid construction and the conjugation donor. The plasmids were constructed using Gibson Assembly. Plasmid extraction kits were purchased from Tiangen Biotech Co., Ltd. (Beijing, China). Plasmids for expression were electroporated into *E. coli* S17-1. DNA fragments were amplified using Q5® High-Fidelity DNA polymerase (New England Biolabs Inc., USA).

For molecular biological studies, *E. coli* was cultured at 37 °C in an LB medium containing (g L^−1^) 10 NaCl (Analytical reagent, Sinopharm Chemical Reagent Co., Ltd., China), 10 tryptone (Analytical reagent, Oxoid, England), and 5 yeast extract (Analytical reagent, Oxoid, England). *H. bluephagenesis* was cultured at 37 °C in a 60-LB medium, namely, the LB medium supplemented with 60 g L^−1^ NaCl. A 20-LB medium indicates the LB medium containing 20 g L^−1^ NaCl. Moreover, 15 g L^−1^ agar (BioDee Biotechnology Co., Ltd., Beijing, China) was added before autoclaving for preparing solid media in Petric plates. Ampicillin (100 μg mL^−1^), chloramphenicol (25 μg mL^−1^), kanamycin (50 μg mL^−1^), or spectinomycin (100 μg mL^−1^), all purchased from BioDee Biotechnology Co., Ltd. (Beijing, China), were added to the above media whenever necessary. Absolute ethanol (Analytical reagent, Beijing Tongguang Chemicals Co., China) and chloroform (Analytical reagent, Beijing Tongguang Chemicals Co., China) were used for PHA extraction and analysis. 3HPA, NADH, and NAD^+^ were purchased from Shanghai Kaiwei Biochemical Co., Ltd. and Abmole (Shanghai, China). Unless indicated otherwise, 1,3-propanediol and all other chemicals were obtained from Shanghai Sangon Biotech Co., Ltd.

### Genome editing using CRISPR/Cas9

Gene insertions and deletions on the chromosome of *H. bluephagenesis* were conducted using CRISPR/Cas9 based on homologous recombination^33,68^. After PCR verification and DNA sequencing, the engineered *H. bluephagenesis* TD with *dddA* gene deleted was designated as *H. bluephagenesis* TDΔ*dddA*. *H. bluephagenesis* TD01 deleted with its native PHA synthesis genes *phaA*, *phaB* and *phaC* is named as *H. bluephagenesis* TDΔ*phaCAB*. *H. bluephagenesis* TDΔ*phaCAB* deleted with *dddA* gene is further named as *H. bluephagenesis* TDΔ*phaCAB*Δ*dddA*. *H. bluephagenesis* TDΔ*phaCAB*Δ*dddA* and *H. bluephagenesis* TD*ΔdddA* inserted with *dhaT*_*Pp*_ and *aldD*_*Pp*_ gene from *Pseudomonas putida* KT2440 under the control of P_Re_ promoter in G4 site on its genome are renamed as *H. bluephagenesis* TD17 and *H. bluephagenesis* TD22, respectively. *H. bluephagenesis* TDΔ*phaCAB*Δ*dddA* and *H. bluephagenesis* TDΔ*dddA* inserted with *adhP* and *aldD*_*Hb*_ gene from *H. bluephagenesis* under the control of P_Re_ promoter on the G4 site are renamed as *H. bluephagenesis* TD25 and *H. bluephagenesis* TD27, respectively.

### Conjugation

Conjugation was performed as an efficient way to transform plasmids into *H. bluephagenesis*^37^. *E. coli* S17-1, with plasmids electro-transformed in, were used as donors. The donors and *H. bluephagenesis* recipient cells were cultured at 37 °C for 4 h till the optical density (OD_600_) reached 0.3–0.5. After collecting 1 mL of cells of *E. coli* S17-1 and *H. bluephagenesis* by centrifugation respectively, the pellet cells were washed twice using a 20-LB medium. Then the cells were re-suspended and mixed at a ratio 1:1, followed by incubation on 20-LB plates at 37 °C for 12 h. The mixed bacteria were spread on plates containing corresponding antibiotics and incubated at 37 °C for 24–48 h for single colonies, which were verified by PCR for future use. *H. bluephagenesis* wild-type and recombinant strains were stored in autoclaved 25% (v/v) glycerol at −80 °C if needed.

### Shake-flask studies

For shake-flask PHA production, a defined minimal medium containing 6% NaCl, 0.05% urea, 0.02% MgSO_4_, 1.0% Na_2_HPO_4_·12H_2_O, 0.15% KH_2_PO_4_, 1.0% trace element solution I, 0.1% trace element solution II and 0.1% yeast extract, was used. Phosphate buffer was changed to 1.4% K_2_HPO_4_·3H_2_O and 0.52% KH_2_PO_4_ in a modified minimal medium. The pH of the solution was adjusted to 8.5–9.0 using 5 M NaOH. Trace element solution I comprises 0.5% Fe (III)-NH_4_-citrate and 0.2% CaCl_2_ dissolved in 1 M HCl; Trace element solution II contains 0.01% ZnSO_4_·7H_2_O, 0.003% MnCl_2_·4H_2_O, 0.03% H_3_BO_3_, 0.02% mg L^−1^ CoCl_2_·6H_2_O, 0.003% NaMoO_4_·2H_2_O, 0.002% NiCl_2_·6H_2_O, and 0.001% CuSO_4_·5H_2_O dissolved in 1 M HCl. Glucose was added at a final concentration of 3% at the beginning of the experiment. While 1,3-propanediol (Analytical reagent, Tianjin Guangfu Chemicals Co., China) was added at 0.5% or other concentrations as indicated.

For all shake-flask studies, the microbial glycerol stocks were resuscitated by streaking on fresh 60-LB plates. Single colonies from streaked or newly-conjugated plates were picked and inoculated in the 60-LB medium for 24 h at 200 rpm to acquire the first seed culture, which was further grown on a fresh 60-LB liquid medium at a volume ratio of 1%. The second seed culture was inoculated for 10 h at 200 rpm until its OD_600_ reached 1.0. Afterward, it was inoculated into 500-mL conical flasks containing 50 mL of the defined minimal medium at a volume ratio of 5% and cultivated for 48 h at 200 rpm. Antibiotics were added if needed. The temperature for all cultivations was 37 °C. All data were analyzed and graphed by GraphPad Prism 8.

### RNA sequencing and quantitative RT-PCR

The bacteria were sampled from shake-flask experiments after 12 h cultivation. Then, the samples were centrifuged at 4000 × *g* and 4 °C for 10 min followed by quick-frozen in liquid nitrogen. For RNA sequencing and analysis, they were transported immediately to the testing company in dry ice (solid carbon dioxide). RNA extraction, sequencing, and analysis were performed by Hangzhou Lianchuan Bio Technologies Co. Ltd. Briefly, RNA was extracted by TRIzol® Reagent (Invitrogen, U.S.A). The RNA library was constructed using Illumina Truseq^TM^ RNA sample prep Kit (Illumina, U.S.A). Sequencing was performed using the Illumina Hiseq platform after the processing of libraries via Truseq SBS Kit v3-HS (Illumina, U.S.A). The expression differences were analyzed using edgeR.

For quantitative RT-PCR, total RNA was isolated from bacterial culture using TRIzol® Reagent according to the manufacturer’s instructions. RNA concentration was determined via spectrophotometry at 260 nm. Removal of genomic DNA and synthesis of cDNA were carried out using PrimeScript RT reagent Kit with gDNA Eraser (Takara). qRT-PCR was conducted using 2× RealStar Green Power Mixture with ROX II (Genstar, China) with the 7500 Fast Real-Time PCR System (Applied Biosystems). Constitutively transcribed 16s rRNA was used as a reference control to normalize the total RNA quantity of different samples. The relative difference of mRNA level was calculated using the ΔΔCt method^69^. Two independent biological samples with three technical repeats for each sample were performed for each qRT-PCR analysis.

### Protein expression and enzyme activities

Plasmids p90, p91, p92, and p98 were constructed encoding genes *adhP*, *aldD*_*Hb*_, *dhaT*_*Pp*_ with a His-tag on C-terminal and *aldD*_*Pp*_ with a His-tag on N-terminal, respectively. The *aldH* gene, with a His-tag on N-terminal, was amplified by PCR using *E. coli* MG1655 genomic DNA as a template and cloned into the p59 plasmid by replacing the *aldD*_*Hb*_ gene. This plasmid was labeled as p99. The *dhaT*_*Kp*_ gene with a His-tag on C-terminal or *puuC* gene with a His-tag on N-terminal was amplified by PCR using *K. pneumoniae* DSMZ 2026 genomic DNA as a template and cloned into the p59 plasmid by replacing the *adhP* or *aldD*_*Hb*_ gene. These plasmids were labeled as p95 and p100. All the recombinant plasmids were conjugated into *H. bluephagenesis* TD*∆dddA* and cultivated in a defined minimal medium containing 20 g L^−1^ glucose, 20 g L^−1^ 1,3-propanediol, and 3 g L^−1^ acetic acid. The recombinant strains were cultured in conical flasks and grown for 24 h. Subsequently, they were harvested by centrifugation under 4000 × *g* and 4 °C for 20 min. Proteins were purified by a His-tag Protein Purification Kit (Beyotime, China) and stored at −80 °C for further usage. The enzyme activity was assayed by a VarioskanFlash plate reader (Version 4.00.53, Thermo Scientific, USA).

The reductive activity (forward reaction) of AdhP, DhaT_Pp_, and DhaT_Kp_ were assayed as follows, respectively: First, the reaction mixture containing 50 mM Tris-HCl buffer (pH 7.0) and 10 nM enzyme was incubated for 2 min at 37 °C. The reaction was initiated by the addition of 0.1 mM of NADH and 5 mM 3HPA. The enzyme activity was determined by measuring the NADH oxidation using the molar extinction coefficient (∆ε_340_) of 6.22 × 10^3^ M^−1^ cm^−1^. The oxidative activity (reverse reaction) of AdhP, DhaT_Pp_, and DhaT_Kp_ were measured as follows: The reaction mixture containing 100 mM potassium carbonate buffer (pH 9.0), 30 mM ammonium sulfate, and 100 nM enzyme was incubated for 2 min at 37 °C. The reaction was initiated by the addition of 2 mM of NAD^+^ and 150 mM 1,3-propanediol^51^. The activity of the aldehyde dehydrogenase was determined by measuring the level of NAD^+^ reduction to NADH at 340 nm. The reaction mixture containing 100 mM potassium phosphate buffer (pH 8.0), 1 mM DTT, and 100 nM enzyme was incubated at 37 °C for 5 min. The reaction was initiated by adding 2.0 mM 3HPA and 2.0 mM NAD^+^. The amount of NADH formed was studied using a molar extinction coefficient (∆ε340) of 6.22 × 10^3^ M^−1^ cm^−1^. One unit of aldehyde dehydrogenase activity was defined as the amount of enzyme needed to reduce 1 μmol of NAD^+^ to NADH in 1 min^49^.

### NADH assays

All cells were harvested after fermentation via centrifugation under 2500 × *g* at 4 °C for 10 min. 200 μL cells were collected from each sample, followed by washing using ice-cold PBS at pH 7.4. The obtained pellet was disrupted by a chemical lysis method using 400 μL BugBuster^®^ Master Mix (ThermoFisher Scientific Inc., USA). All operations were strictly conducted based on instructions of NAD/NADH Assay Kit S0175 (Beyotime, China).

### 3HP and PHB production in a 7 L fermenter

Fed-batch studies were performed in a 7 L *BioFlo* fermenter (New Brunswick Scientific, U.S.A) with a 3 L working volume without sterilization. The growth temperature was 37 °C, maintained via a cooling circulation pump (Henan Jinghua Instrument, China). The pH was maintained at 8.5 using an automatic pump added 5 M NaOH into the culture system when needed. The fermentation agitation speed was set at 200 rpm at the beginning, coupled with dissolved oxygen (DO) concentration, and gradually increased to 800 rpm to maintain the DO level above 30% compared with the one in a cell-free fermenter (set as 100%). The agitation speed was maintained at 800 rpm to the end of fermentation. The inlet airflow rate was maintained at 3 L min^−1^ during the entire fermentation process.

The seed culture has the same medium as that in shake flasks. A 300 mL seed culture (10% of the total volume) was prepared for inoculation into the fermenter. The OD_600_ of the seeds was adjusted to 3. For fermentor studies, low-cost substrates were used and the defined minimal medium was modified by utilizing 16 g L^−1^ corn steep (Baisheng, Shandong Biotechnology Co., Ltd., China) instead of the expensive yeast extract, the NaCl concentration was reduced to 40 from 60 g L^−1^.

For 3HP production, a two-stage and intermittent feeding strategy were adopted using different feeding solutions. 400 mL feeding solution I containing 800 g L^−1^ glucose and 30 g L^−1^ urea was used to increase cell mass accumulation and PHB formation during the first 16 h. 200 mL feeding Solution II containing 800 g L^−1^ glucose and 15 g L^−1^ urea was added after feeding Solution I exhausted. The flow rates of the feeding solutions depended on the residual glucose concentrations that should be controlled between 5 and 10 g L^−1^ measured using the GA-3 blood glucose meter and GA-3 blood glucose test strip (Sinocare, China). 60 g L^−1^ 1,3-propanediol and 6 g L^−1^ acetic acid were added three times separately at 24, 32, and 38 h after the beginning of the fermentations. OD_600_ of the cultured bacteria was measured by a V-5600 visible light spectrophotometer (Shanghai Yuanxi Instrument, China).

For P3HB3HP production, a two-stage feeding strategy was used with different feeding solutions. 400 mL feeding solution I containing 800 g L^−1^ glucose, 30 g L^−1^ urea, and 75 g L^−1^ 1,3-propanediol was used to increase cell mass and P3HB3HP formation during the first 20 h. 200 mL feeding solution II containing 800 g L^−1^ glucose, 15 g L^−1^ urea, and 75 g L^−1^ 1,3-propanediol was added after feeding solution I exhausted.

### PHA purification and analysis

After shake-flask growth, cells were harvested by centrifugation (CR21 GIII, Hitachi, Japan) at 8000 × *g* for 10 min, washed twice with distilled water, and placed in −80 °C for 4 h. After cells lyophilizing for 12 h, their intracellular PHA was extracted using a Soxtec 2050 Soxhlet extractor (Foss, Denmark). PHA polymers were purified by re-dissolving the extracted materials in chloroform, then precipitated using absolute ethanol, centrifuged at 12,000 × *g* for 10 min to harvest the purified PHA materials, followed by lyophilization again to remove residual ethanol.

The dry cell mass was determined by weighing 30 mL lyophilized centrifuged bacterial cells. For the esterification reaction, 40–60 mg lyophilized cells were placed into a 2 mL esterification reagent containing 3% sulfuric acid and 0.1% benzoic acid, both dissolved in absolute methanol. After adding 2 mL chloroform, the whole reaction was performed at 100 °C for 4 h. PHA contents were determined by GC-2014 gas chromatography (Shimadzu, Japan). P3HB (99.9%, Sigma-Aldrich, Germany) was used as standards, respectively. The peak of benzoic acid was regarded as the internal standard.

### Chemical quantification using high-performance liquid chromatography

Concentrations of 3HP, glucose, acetic acid, and 1,3-propanediol were determined via high-performance liquid chromatography (HPLC). An LC-20 instrument (Shimadzu, Japan) with an Aminex HPX-87H column (Bio-Rad, U.S.A) and a RID-10A refractive index detector (Shimadzu) was employed. The mobile phase was a 5 mM degassed H_2_SO_4_ with a flow rate of 0.5 mL min^−1^. The column temperature was maintained at 55 °C^70^. The culture media were centrifuged at 12,000 × *g* and 4 °C for 2 min to obtain the supernatant, which was then filtered through a 0.22 μm polyethersulfone membrane syringe filter (Jinglong, China) and used as the samples with a loading volume of 30 μL. Standards of the above chemicals were prepared by five different concentrations to draw the standard curves, from which the concentrations of the sample were calculated.

### Nuclear magnetic resonance

PHA samples (2–5 mg) were dissolved in deuterated chloroform for ^1^H NMR with a JNM-ECA600 nuclear magnetic resonance instrument (JEOL, Japan). Chemical shifts were given in ppm while 0.03% v/v tetramethylsilane (TMS) served as an internal standard to calibrate against the signal of samples.

### Reporting summary

Further information on research design is available in the Nature Research Reporting Summary linked to this article.

## Supplementary information

Supplementary Information

Reporting Summary

Description of Additional Supplementary Files

Supplementary Data 1

Supplementary Data 2

Supplementary Data 3

## Data Availability

RNA-seq data that support the findings of this study have been deposited in the Gene Expression Omnibus (GEO) with the accession code GSE159233. The DhaT_Pp_, AldD_Pp_, DddA, AldD_Hb_ and AdhP sequences used in this study are under the accession numbers AAN68411.1, AAN66172.1, WP_009721523.1, WP_009721344.1 and WP_039868491.1, respectively. Data supporting the findings of this work are available within the paper and its supplementary information files. A reporting summary for this article is available as a supplementary information file. All relevant data are available from the authors upon reasonable request. Source data are provided with this paper.

## Source data

Source Data
