# Supplementary material for: Hyperproduction of 3-hydroxypropionate by Halomonas bluephagenesis
Source: Nat Commun. 2021 Mar 8;12:1513. doi: 10.1038/s41467-021-21632-3 (PMC7940609; doi:10.1038/s41467-021-21632-3)
Supplement: Supplementary file 1 — Supplementary Information [file 41467_2021_21632_MOESM1_ESM.pdf]

Supplementary information

Hyperproduction of 3-hydroxypropionate by *Halomonas bluephagenesis*

Jiang *et al*.

Supplementary Figures

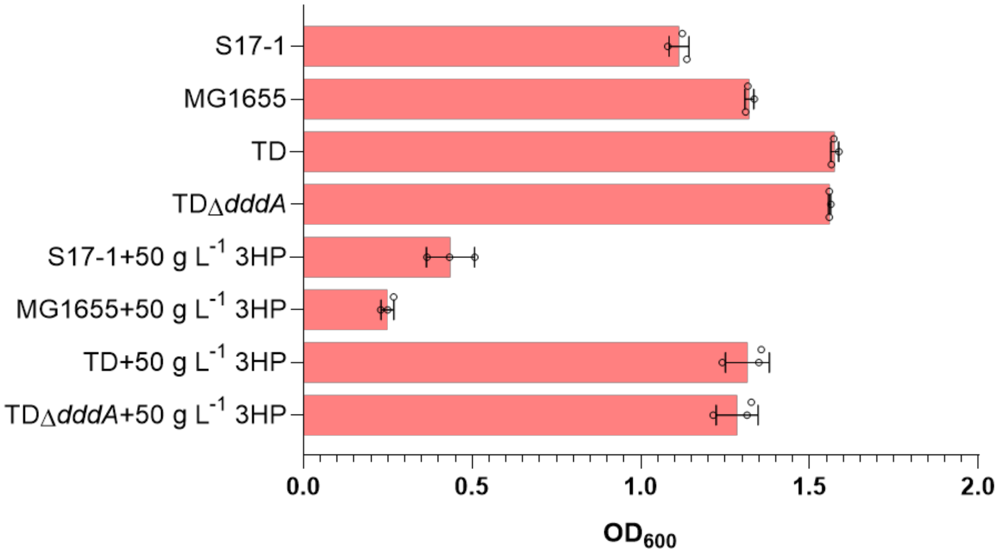

Supplementary Figure 1. The 3HP tolerance of *E. coli* and *H. bluephagenesis*. *E. coli* S17-1 and MG1655 were grown in microtiter plates added with 200 µl LB medium containing 0 and 50 g L^{-1} 3HP, respectively. *H. bluephagenesis* TD and TDΔ*dddA* were grown in microtiter plates added with 200 µl 60LB medium containing 0 and 50 g L^{-1} 3HP, respectively. All the samples were cultured at 37°C, pH=7, 600 rpm for 48 h. All data represent the mean of n=3 biologically independent samples and error bars show standard deviations.

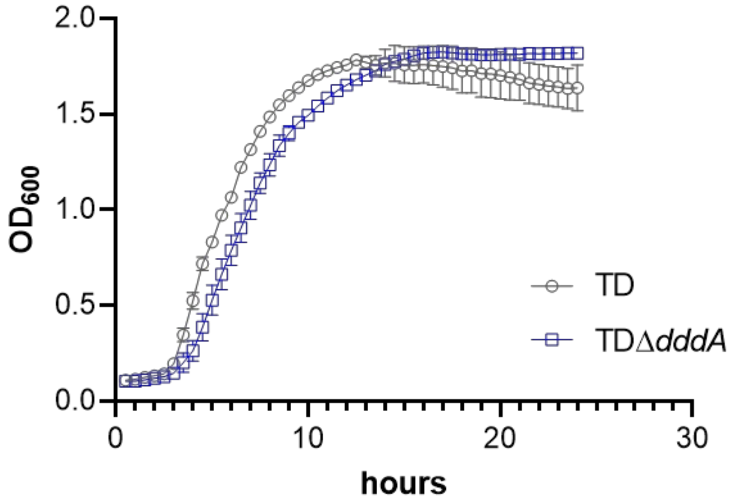

Supplementary Figure 2. Growth of wild type *H. bluephagenesis* TD and *dddA* gene deleted *H. bluephagenesis* TD measured as OD_{600}. Cells were grown in microtiter plates added with 200 μl 60LB medium. All data represent the mean of n=5 biologically independent samples and error bars show standard deviations.

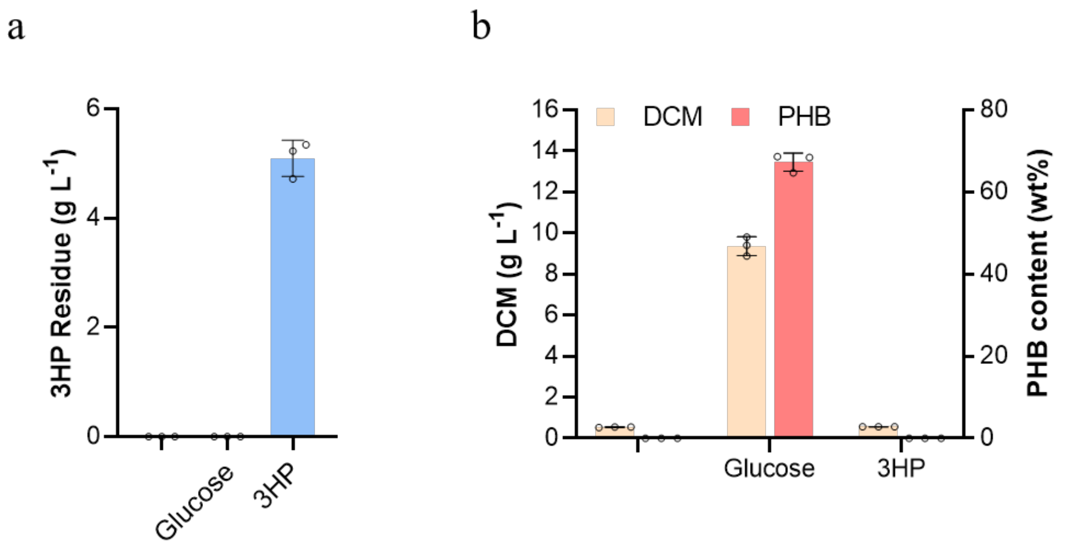

Supplementary Figure 3. Identification of the inactivation of 3HP degradation ability in *H. bluephagenesis* TDΔ*dddA*. (**a**) Residual 3HP and (**b**) PHB production by *H. bluephagenesis* TDΔ*dddA* in the presence of 30 g L^{-1} glucose or 5 g L^{-1} 3HP, respectively. All titers were obtained after 48 h cultivation at 200 r.p.m. and 37°C. The initial pH of all shake flask studies was 9. All data represent the mean of n=3 biologically independent samples and error bars show s.d.

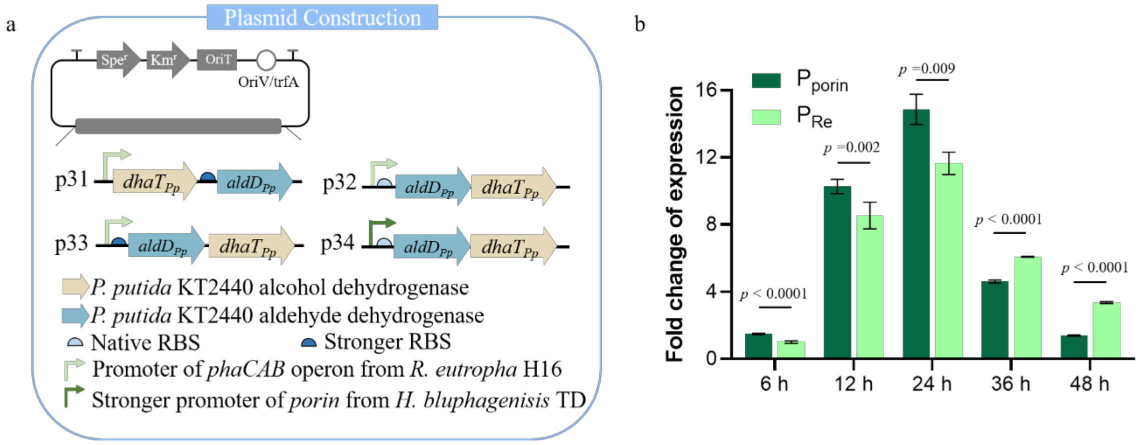

Supplementary Figure 4. Manipulation of expression level of the *aldD*$_{Pp}$ gene. a. A stronger RBS was cloned instead of the original p30 to construct plasmid p31. The order of *aldD*$_{Pp}$ and *dhaT*$_{Pp}$ operon was changed to construct p32. The order of *aldD*$_{Pp}$ with a stronger RBS and *dhaT*$_{Pp}$ operon was changed to construct p33. A stronger promoter was cloned instead of the original p32 to construct plasmid p34. **b.** qRT-PCR analysis of *aldD*$_{Pp}$ expression levels under the control of P_{Porin} and P_{Re}. Both recombinants were cultured in shake flasks and samples at 6, 12, 24, 36 and 48 h were prepared. All data represent the mean of n=3 biologically independent samples and error bars show s.d. Two-tailed Student's t tests were performed to determine the statistical significance for two comparison groups.

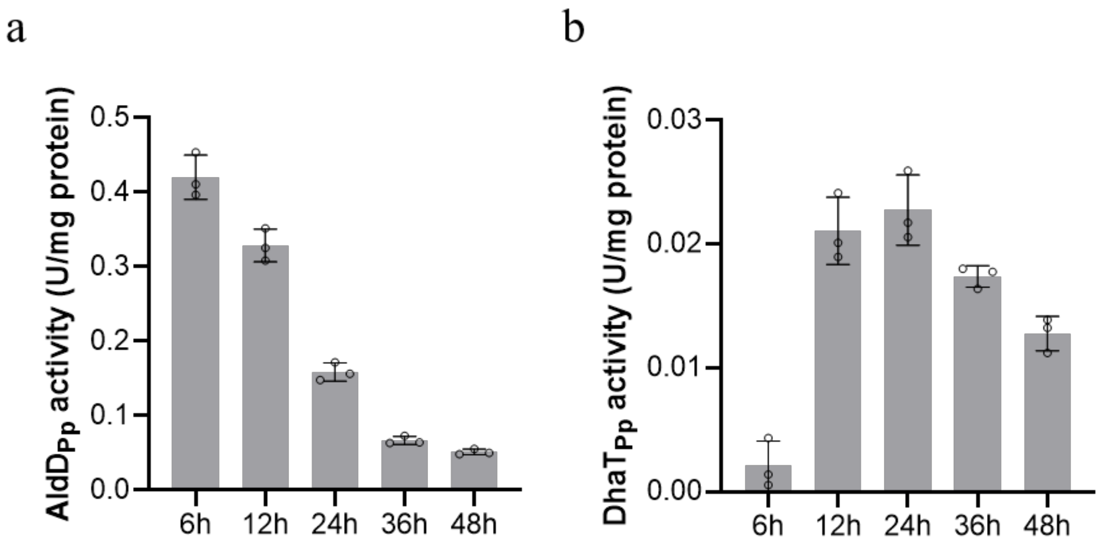

Supplementary Figure 5. AldD$_{Pp}$ (a) and DhaT$_{Pp}$ (b) activities measured over time in crude extracts. *H. bluephagenesis* TDΔ*dddA* overexpressing DhaT$_{Pp}$ or AldD$_{Pp}$ was cultivated in the 60LB medium. The recombinants were cultured in conical flasks and grown for 6, 12, 24, 36 and 48 h, respectively. All data represent the mean of n=3 biologically independent samples and error bars show s.d.

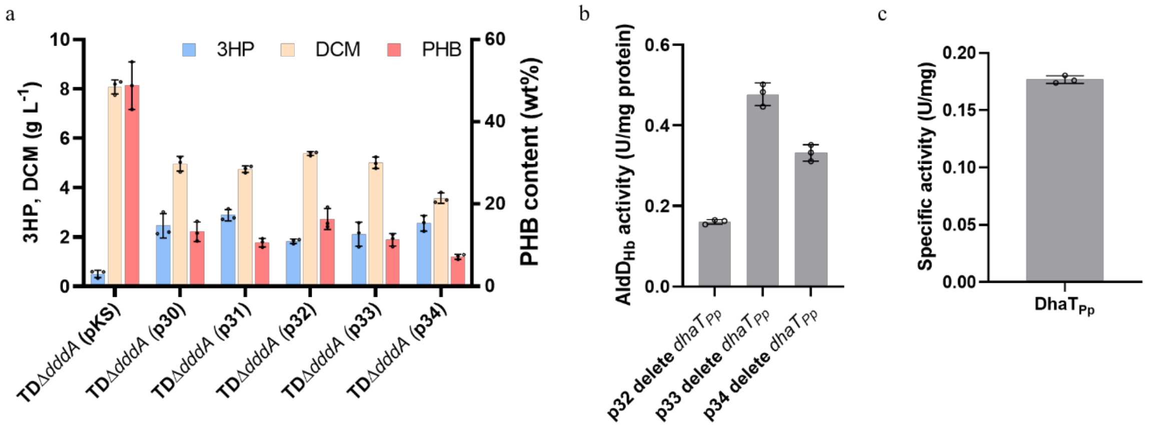

Supplementary Figure 6. Effects of manipulating expression levels of the *$aldD_{Pp}$* gene on production of 3HP and the comparation of enzyme activities. a. Cells were grown in the defined minimal medium containing 30 g L^{-1} glucose and 10 g L^{-1} 1,3-propanediol. All titers were obtained after 48 h cultivation at 200 r.p.m. and 37°C. The initial pH of all shake flask studies was 9. **b**. The enzyme activity of $AldD_{Pp}$ was assayed using the recombinant *H. bluephagenesis* TDΔ*dddA* harboring p32-p34 inactivated the *$dhaT_{Pp}$* gene cultivated in 60LB medium for 24 h. The comparation of activities was conducted using crude extract. **c**. The specific activity of $DhaT_{Pp}$ oxidative activity in purified enzyme. All data represent the mean of n=3 biologically independent samples and error bars show s.d.

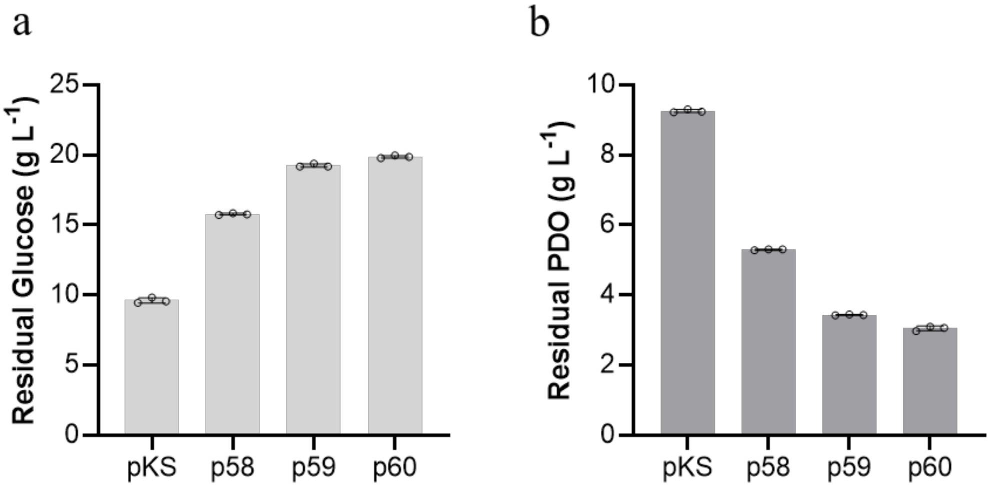

Supplementary Figure 7. Residual glucose (a) and 1,3-propanediol (b) in cultures of *H. bluephagenesis* TDΔ*dddA* harboring different plasmids, respectively. Cells were grown in the defined minimal medium containing 30 g L^{-1} glucose, 10 g L^{-1} 1,3-propanediol and 3 g L^{-1} acetic acid. All titers were obtained after 48 h cultivation at 200 r.p.m. and 37°C. The initial pH of all shake flask studies was 9. All data represent the mean of n=3 biologically independent samples and error bars show s.d.

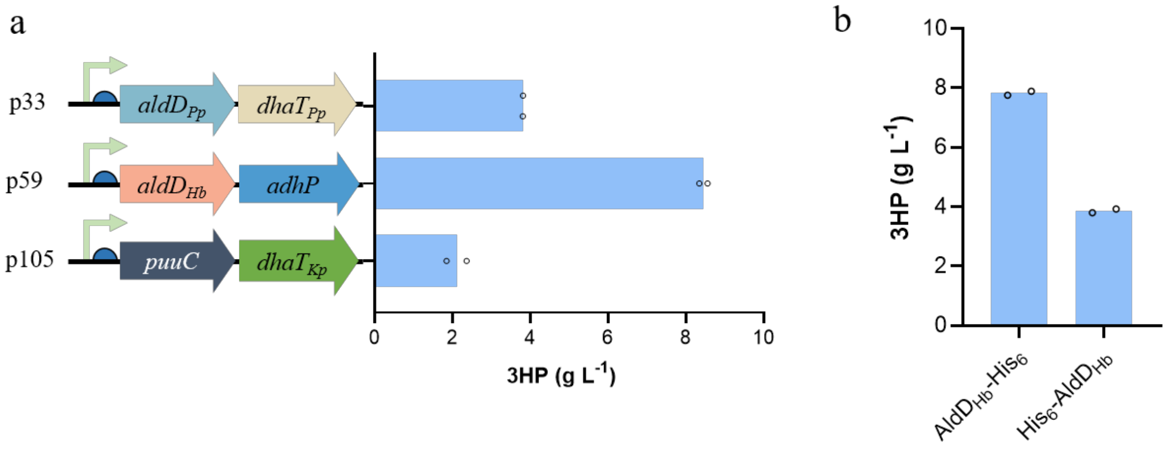

Supplementary Figure 8. 3HP productions using enzymes from different species, respectively. 3HP production using enzymes from different species (**a**) and AldD$_{Hb}$ with different His$_6$-tag locations (**b**). Cells were grown in the defined minimal medium containing 20 g L^{-1} glucose, 20 g L^{-1} 1,3-propanediol and 3 g L^{-1} acetic acid. All titers were obtained after 48 h cultivation at 200 r.p.m. and 37° C. The initial pH of all shake flask studies was 9. All data represent the mean of n=2 biologically independent samples.

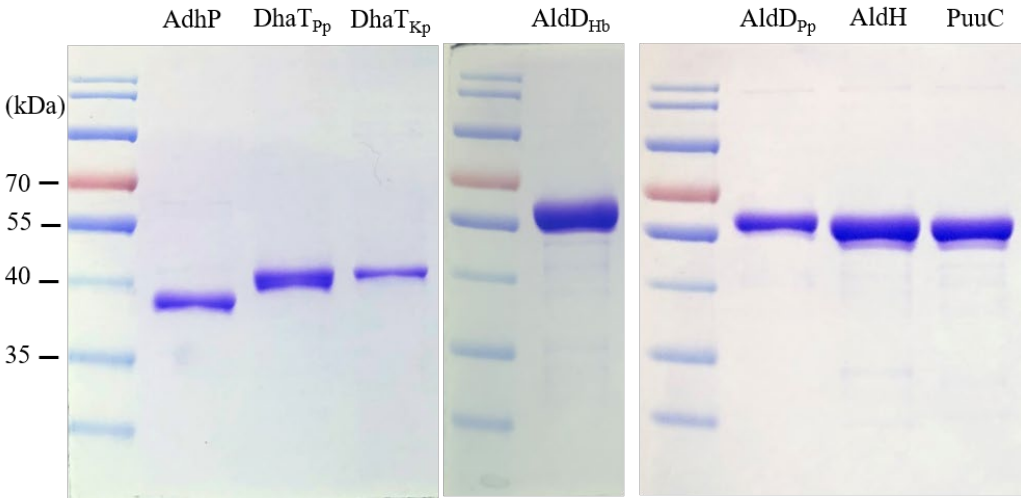

Supplementary Figure 9. SDS-PAGE analysis of recombinant alcohol dehydrogenases (AdhP, $DhaT_{Pp}$ and $DhaT_{Kp}$) and aldehyde dehydrogenases ($AldD_{Hb}$, $AldD_{Pp}$, AldH and PuuC). The purified His_6-tagged enzymes were analyzed in a 12% denatured SDS-PAGE gel. A depiction of one of three independent SDS/PAGE analyses.

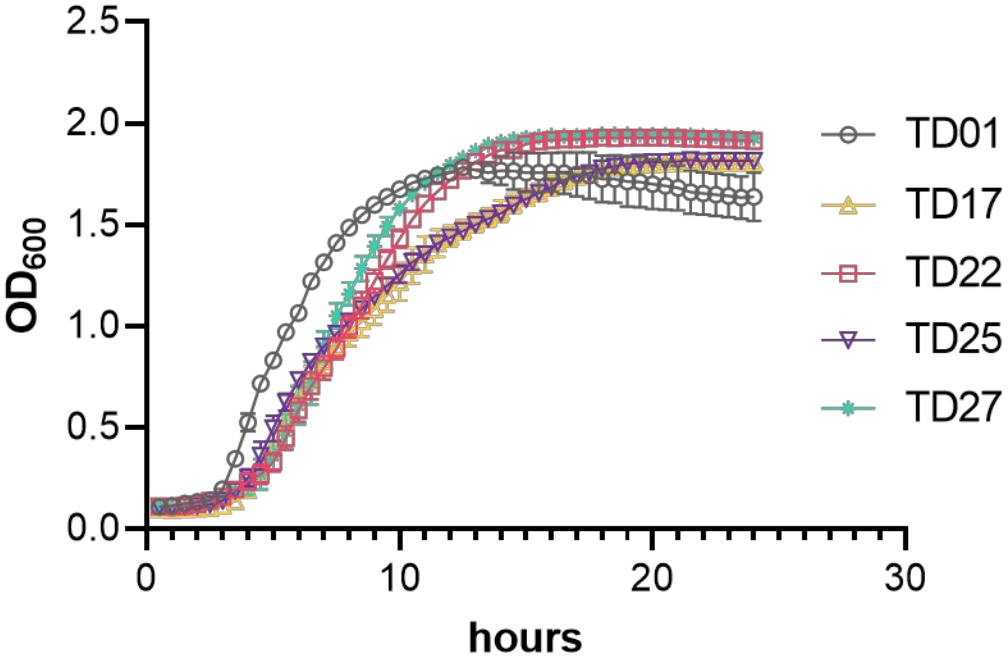

Supplementary Figure 10. Growth of aldehyde dehydrogenase and alcohol dehydrogenase genome-expressing recombinants *H. bluephagenesis*. Cells were grown in microtiter plates containing 200 µl 60LB medium in each well. All data represent the mean of n=5 biologically independent samples, error bars show standard deviations.

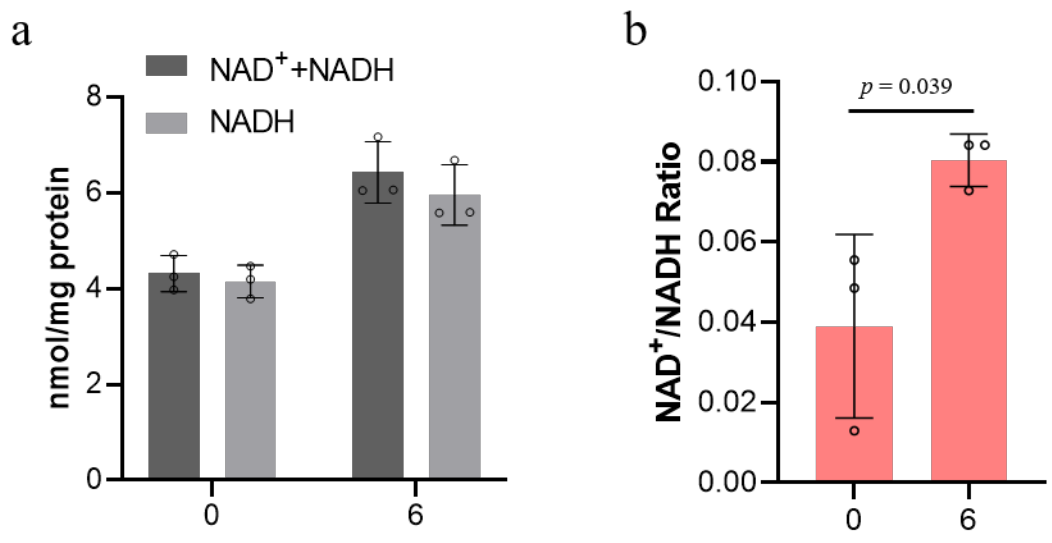

Supplementary Figure 11. Studies on NADH and NAD$^+$ in *H. bluephagenesis* during the co-production of 3HP and PHB in the presence and absence of acetic acid, respectively. Total NADH, NAD$^+$ + NADH (**a**) and NAD$^+$/NADH (**b**) ratio of *H. bluephagenesis* TD27 grown in the modified minimal medium containing 20 g L^{-1} glucose and 20 g L^{-1} 1,3-propanediol, in the presence and absence of 6 g L^{-1} acetic acid for 48 h. All data represent the mean of n=3 biologically independent samples and error bars show s.d. Two-tailed Student's t tests were performed to determine the statistical significance for two group comparisons.

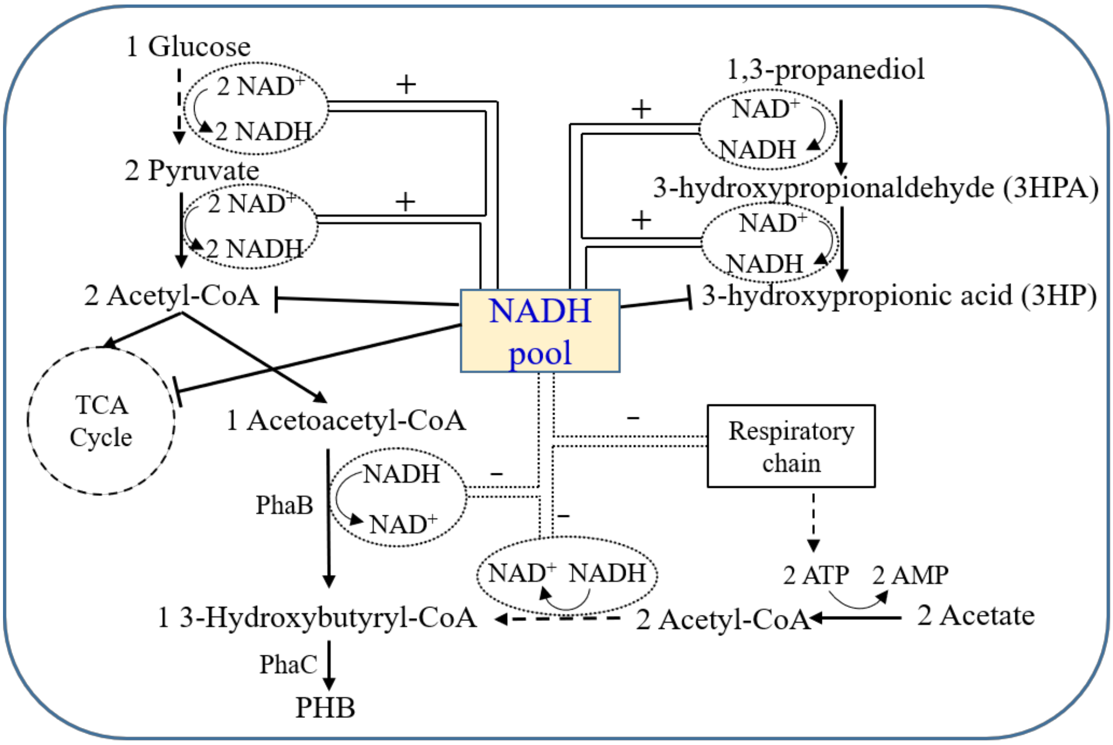

Supplementary Figure 12. Metabolic pathways of PHB and 3HP synthesis in engineered *H. bluephagenesis*. PHB was synthetized directly from acetyl-CoA via NADH dependent fermentative pathway. The NADH-excess condition resulted from 3-HP formation via 1,3-propanediol oxidation can be relieved by accelerating PHB production via a bigger acetyl-CoA pool, improving not only PHA synthesis but also glycolysis, TCA cycle and 3HP synthesis.

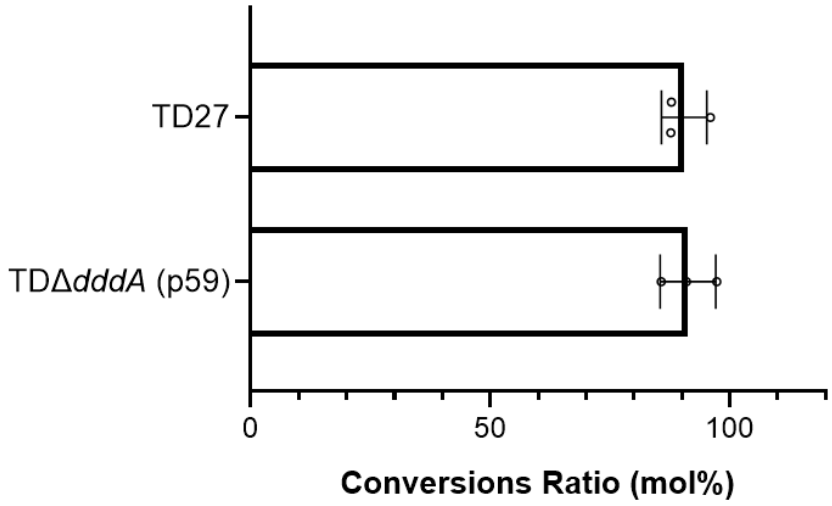

Supplementary Figure 13. Conversion of 1,3-Propanediol to 3HP by *H. bluephagenesis* TD27. 1,3-propanediol conversion efficiency=3HP (mol L^{-1})/consumed 1,3-propanediol (mol L^{-1}). All data represent the mean of n=3 biologically independent samples and error bars show s.d.

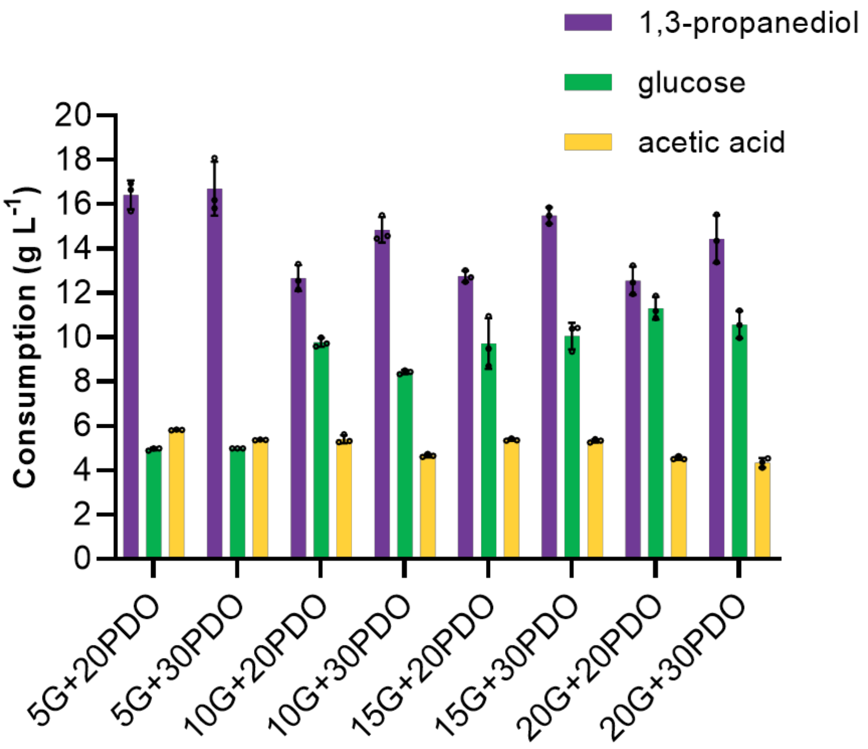

Supplementary Figure 14. Consumption ratios of 1,3-propanediol: glucose: acetic acid for cultures of *H. bluephagenesis* TD27. 5, 10, 15 and 20 g L^{-1} glucose (5G, 10G, 15G and 20G) were co-fed with 20 g L^{-1} 1,3-propanediol (20PDO) or 30 g L^{-1} 1,3-propanediol (30PDO), respectively, to cultures of *H. bluephagenesis* TD27 in a modified minimal medium containing 6 g L^{-1} acetic acid. All data represent the mean of n=3 biologically independent samples and error bars show s.d.

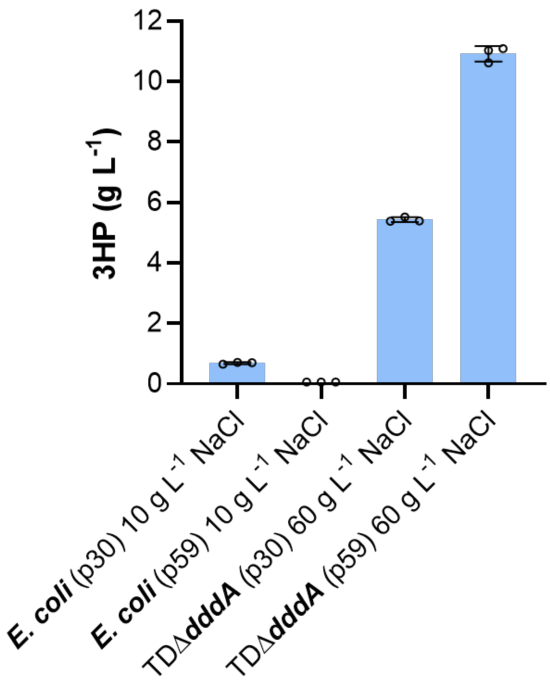

Supplementary Figure 15. Comparison of 3HP production by *E. coli* and *H. bluephagenesis* TDΔ*dddA*. Cells were grown in the modified minimal medium with 20 g L^{-1} glucose, 20 g L^{-1} 1,3-propanediol and 6 g L^{-1} acetic acid. *E. coli* was cultured in a modified minimal medium containing 1% NaCl, and *H. bluephagenesis* in another modified minimal medium containing 6% NaCl. All titers were obtained after 48 h cultivation at 200 r.p.m. and 37°C. All data represent the mean of n=3 biologically independent samples and error bars show s.d.

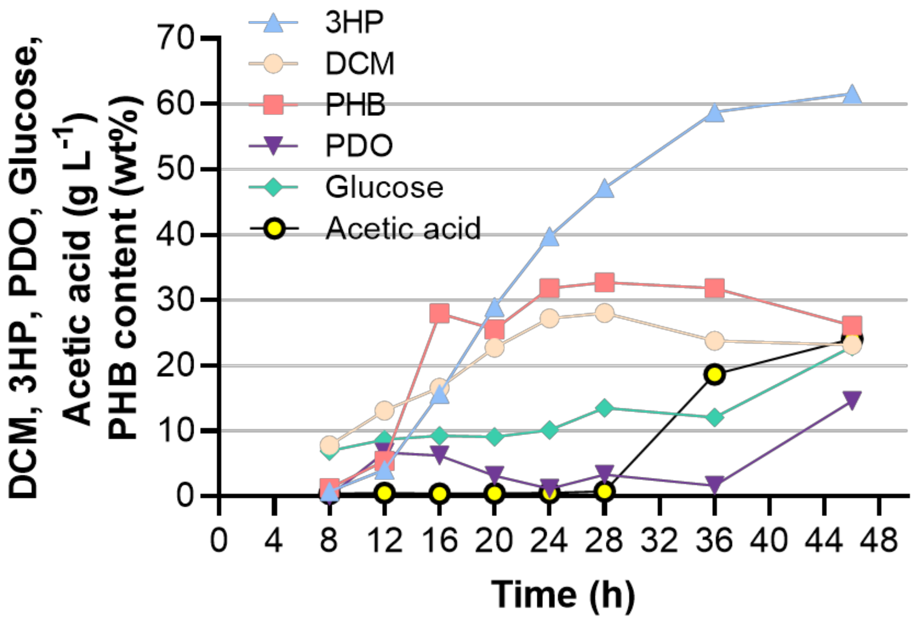

Supplementary Figure 16. Fed-batch fermentation using a continuous feeding strategy. Time profiles of cell growth (CDW), PHB accumulation and residual concentrations of carbon sources (glucose, 1,3-propanediol, acetic acid) and 3HP formation during the fed-batch culture of *H. bluephagenesis* TD27. 1,3-propanediol and glucose were added at 8 h under the continuous feeding strategy.

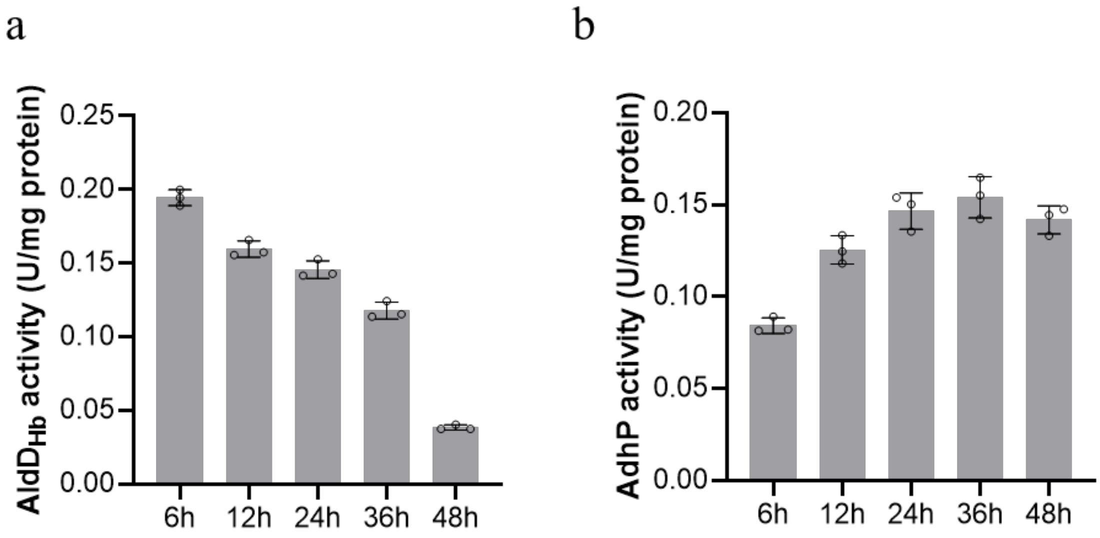

Supplementary Figure 17. AldD$_{Hb}$ (a) and AdhP (b) activities measured over time in crude extracts. *H. bluephagenesis* TDΔ*dddA* overexpressing AdhP or AldD$_{Hb}$ was cultivated in the 60LB medium. The recombinants were cultured in conical flasks and grown for 6, 12, 24, 36 and 48 h, respectively. All data represent the mean of n=3 biologically independent samples and error bars show s.d.

a

b

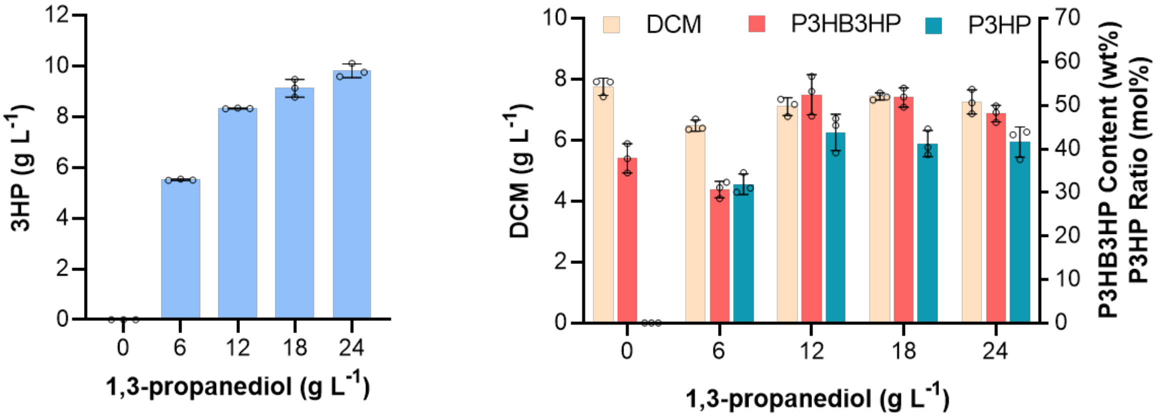

Supplementary Figure 18. Quantitation of 3HP and its copolymers produced by metabolically engineered *H. bluephagenesis* TD27 overexpressing *pcs*. (**a**) 3HP production and (**b**) P3HB3HP contents and monomer compositions produced by engineered *H. bluephagenesis* TD27 overexpressing *pcs* grown in the presence of 0, 6, 12,18 and 24 g L^{-1} 1,3-propanediol. b. For the shake flask cultivation, cells were grown in the defined minimal medium added with 20 g L^{-1} glucose and 6 g L^{-1} acetic acid as carbon source. All titers were obtained after 48 h cultivation at 200 r.p.m. and 37°C. The initial pH of all shake flask studies was 9. All data represent the mean of n=3 biologically independent samples and error bars show s.d.

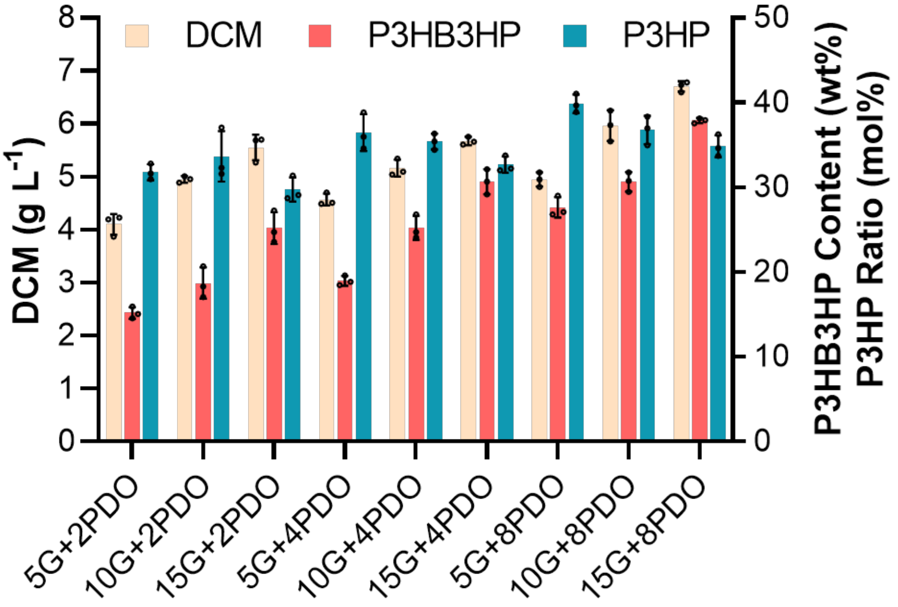

Supplementary Figure 19. Effects of glucose and 1,3-propanediol concentration on the accumulations of P3HB3HP and its 3HP ratio in the copolymer produced by *H. bluephagenesis* TD27 (p129). 5, 10 and 15 g L^{-1} glucose (5G, 10G and 15G) were co-fed with gradient concentrations of 1,3-propanediol (2, 4 and 8 g L^{-1}) (2PDO, 4PDO and 8PDO), respectively, for growth of *H. bluephagenesis* TD27 (p129). Cells were grown in the modified minimal medium with 6 g/L acetic acid as carbon source and different concentrations of glucose and 1,3-propanediol, respectively. All titers were obtained after 48 h cultivation at 200 r.p.m. and 37° C. The initial pH of all shake flask studies was 9. All data represent the mean of n=3 biologically independent samples and error bars show s.d.

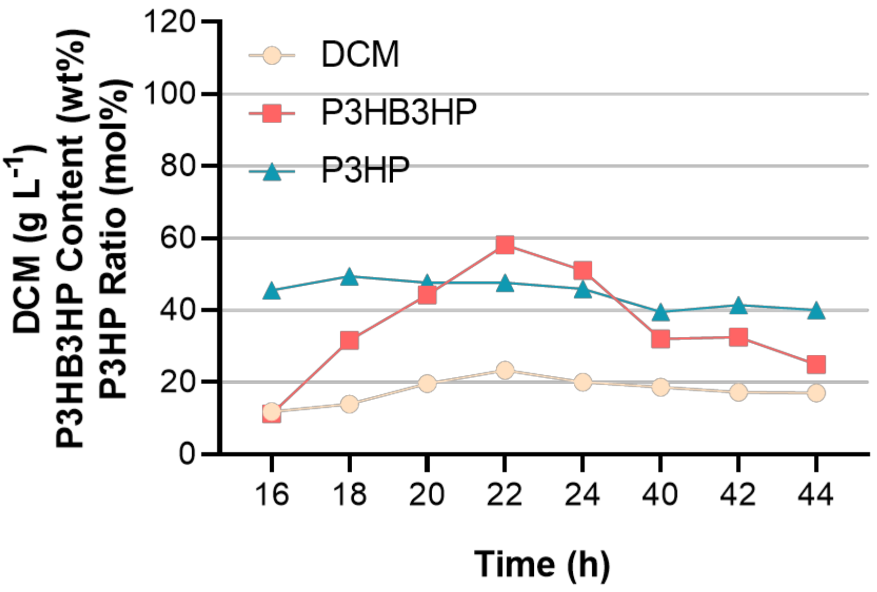

Supplementary Figure 20. Fed-batch fermentation for P3HB3HP production by *H. bluephagenesis* TD 27 (p129). Time profiles of cell growth (CDW), P3HB3HP content and P3HP ratio during the fed-batch cultures of *H. bluephagenesis* TD 27 (p129). 400 ml feed solution I contain 800 g L^{-1} glucose, 4 g L^{-1} urea and 75 g L^{-1} 1,3-propanediol was used to increase cell mass and P3HB3HP formation during the first 20 h. 200 ml feed Solution II containing 800 g L^{-1} glucose, 15 g L^{-1} urea and 75 g L^{-1} 1,3-propanediol was added after fed Solution I exhausted.

Supplementary Tables

Supplementary Table 1. *H. bluephagenesis* strains used in this study.

strain	Descriptions	Source
E. coli S17-1	*tra* genes of RP4 plasmid integrated on the chromosome; *recA*, *proA*, *thi-1, endA1*	[1]
H. bluephagenesis TD	wild-type strain	[2]
H. bluephagenesis TDΔ*dddA*	*H. bluephagenesis* TD deleted *dddA* gene	This study
H. bluephagenesis TDΔ*phaCAB*Δ*dddA*	*H. bluephagenesis* TD deleted *phaC*, *phaA*, *phaB* and *dddA* gene	This study
H. bluephagenesis TD17	*H. bluephagenesis* TDΔ*phaCAB*Δ*dddA* with RE promoter, $dhaT_{Pp}$ and $aldD_{Pp}$ gene from *P. putida* inserted into genome	This study
H. bluephagenesis TD22	*H. bluephagenesis* TDΔ*dddA* with RE promoter, $dhaT_{Pp}$ and $aldD_{Pp}$ gene from *P. putida* inserted into genome	This study
H. bluephagenesis TD25	*H. bluephagenesis* TDΔ*phaCAB*Δ*dddA* with RE promoter, *adhP* and $aldD_{Hb}$ gene inserted into genome	This study
H. bluephagenesis TD27	*H. bluephagenesis* TDΔ*dddA* with RE promoter, *adhP* and $aldD_{Hb}$ gene inserted into genome	This study

Supplementary Table 2. Plasmids used in this study.

Plasmids	Descriptions	Source
pSEVA321	An expression vector, *trfA* replication origin, *oriT*, Cm^R	[3]
pSEVA341	pRO1600/ColE1 replication origin, *oriT*, Cm^R	[3]
pKS	An pSEVA321 derived vector, Kan^R and Spe^R	This study
p30	pKS-pRe-*dhaT*-native RBS-*aldD*	This study
p31	pKS-pRe-*dhaT*-synthetic stronger RBS-*aldD*	This study
p32	pKS-pRe-native RBS-*aldD-dhaT*	This study
p33	pKS-pRe-synthetic stronger RBS-*aldD-dhaT*	This study
p34	pKS-pPorin-native RBS-*aldD-dhaT*	This study
p55	pKS-pRe-native RBS-$aldD_{Hb}$-*GME_RS01345*	This study
p56	pKS-pRe-native RBS-$aldD_{Hb}$-*GME_05160*	This study
p57	pKS-pRe-native RBS-$aldD_{Hb}$-*GME_RS01585*	This study
p58	pKS-pRe-native RBS-$aldD_{Hb}$-*GME_RS00365*(*adhP*)	This study
p59	pKS-pRe-synthetic stronger RBS-$aldD_{Hb}$-*adhP*	This study
p60	pKS-pPorin-native RBS-$aldD_{Hb}$-*adhP*	This study
p129	pKS-pPorin-$phaC_{Re}$-*pcs*	This study
p130	pKS-pPorin-$phaC_{Re}$-*pduP*	This study
p131	pKS-pPorin-$phaC_{Re}$-*prpE*	This study
p132	pKS-pPorin-$phaC_{Re}$-*acoE*	This study
p90	pKS-pRe-synthetic stronger RBS-$aldD_{Hb}$-*adhP-Histag*	This study
p92	pKS-pRe-synthetic stronger RBS-$aldD_{Pp}$-$dhaT_{Pp}$-*Histag*	This study
p95	pKS-pRe-synthetic stronger RBS-$aldD_{Hb}$-$dhaT_{Kp}$-*Histag*	This study
p91	pKS-pRe-synthetic stronger RBS-$aldD_{Hb}$-*Histag-adhP*	This study
p98	pKS-pRe-synthetic stronger RBS-*Histag*-$aldD_{Pp}$-$dhaT_{Pp}$	This study
p99	pKS-pRe-synthetic stronger RBS-*Histag-aldH-adhP*	This study
p100	pKS-pRe-synthetic stronger RBS-*Histag-puuC-adhP*	This study
p105	pKS-pRe-synthetic stronger RBS-*puuC*- $dhaT_{Kp}$	This study

Supplementary Table 3. Kinetic parameters of PuuC.

Enzyme	K_m (mM)	K_{cat} (s^{-1})	K_{cat}/K_m ($s^{-1}mM^{-1}$)
PuuC	9.38±0.24	6.32±0.15	0.67±0.004

Assay conditions: 50 mM, potassium phosphate buffer (pH 8.0) containing 1.0 mM DTT; 2 mM, NAD^+; 11.7 μg ml^{-1}, PuuC; 3HPA concentration was varied from 0.02 to 15 mM; Temperature was set at 37ºC. Each value represents the mean ± standard deviation in triplicate experiments.

Supplementary Table 4. The glucose used during co-production of 3HP and PHB in the presence and absence of acetic acid.

Cultures	glucose consumption ($g\ L^{-1}$)	acetic acid consumption ($g\ L^{-1}$)	3HP ($g\ L^{-1}$)	DCM ($g\ L^{-1}$)	PHB ($g\ L^{-1}$)
TD25+20G+20PDO	8.82±0.45	0	4.24±0.07	ND	0
TD27+20G+20PDO	5.84±0.23	0	6.39±0.08	3.68±0.07	0.21±0.05
TD27+20G+20PDO+6AA	10.50±0.60	3.99±0.26	9.27±0.61	7.98±0.36	3.18±0.24

H. bluephagenesis TD25: no PHB production, TD25 cultured in the minimal medium containing 20G (20 g L^{-1} glucose) and 20PDO (20 g L^{-1} 1,3-propanediol); TD27+20G+20PDO: PHB production from glucose; TD27+20G+20PDO+6AA: PHB production from glucose and acetic acid, 6AA (6 g L^{-1} acetic acid). ND: not detected. Each value represents the mean ± standard deviation in triplicate experiments.

Supplementary Table 5. Promoter and RBS sequence used in this study.

Name	Sequence (5’-3’)
P_{Porin} promoter	cggccgctgagacctgccagttgcccatgggttccttaaaaaaatgcaaatcgta<br>aaaaaacactgtttttttctattgcgttcactggaatcccagtatagagtttgacctgc<br>gagcattggactataacaaggcttagttgaggacatgccgcataccaccgggaa<br>aaaccggaggatggcataaagagcatggcccgcaagccagctgcagactt
P_{Re} promoter	gatgcgagcgctgcataccgtccggtaggtcgggaagcgtgcagtgccgagg<br>cggattcccgcattgacagcgcgtgcgttgcaaggcaacaatggactcaaatgt<br>ctcggaatcgctgacgattcccaggtttctccggcaagcatagcgcatggcgtct<br>ccatgcgagaatgtcgcgcttgccggataaaaggggagccgctatcggaatgg<br>acgcaagccacggccgcagcaggtgcggtcgagggcttccagccagttccag<br>ggcagatgtgccggcagaccctcccgctttgggggaggcgcaagccgggtcc<br>attcggatagcatctccccatgcaaagtgccggccagggcaatgcccggagcc<br>ggttcgaatagtgacggcagagagacaatcaaataggaggtcgagcta
Stronger RBS	cctgtagaaataattttgtttaactttaataaggagatatacc
gRNA of *dddA* gene deletion	agtgctagacgatttcgccg
gRNA of G4 site gene insertion	ttcacctagctagatgagac

Supplementary References

1 Simon, R., Priefer, U. & Pühler, A. A broad host range mobilization system for in vivo genetic engineering: transposon mutagenesis in gram negative bacteria. *Biotechnology. (N. Y.)* **1**, 784-791 (1983).

2 Tan, D., Wu, Q., Chen, J. C. & Chen, G. Q. Engineering *Halomonas* TD01 for the low-cost production of polyhydroxyalkanoates. *Metab. Eng.* **26**, 34-47 (2014).

3 Silva-Rocha, R. *et al.* The Standard European Vector Architecture (SEVA): a coherent platform for the analysis and deployment of complex prokaryotic phenotypes. *Nucleic Acids Res.* **41**, D666-D675 (2013).
