# Supplementary material for: Hyperproduction of 3-hydroxypropionate by Halomonas bluephagenesis
Source: Nat Commun. 2021 Mar 8;12:1513. doi: 10.1038/s41467-021-21632-3 (PMC7940609; doi:10.1038/s41467-021-21632-3)
Supplement: Supplementary file 3 — Description of Additional Supplementary Files [file 41467_2021_21632_MOESM3_ESM.pdf]

Description of Additional Supplementary Files

File Name: Supplementary Data 1
Description: The primers used in this study.

File Name: Supplementary Data 2
Description: The differentially expressed genes when the cells grown under culture conditions with and without 3HP as the carbon source.

File Name: Supplementary Data 3
Description: The differentially expressed genes when the cells grown with or without 1,3-propanediol in culture media containing 30 g L-1 glucose.
